# The gestural repertoire of Bwindi mountain gorillas (*Gorilla beringei beringei*): gesture form and frequency of use

**DOI:** 10.1007/s10071-025-01977-8

**Published:** 2025-07-29

**Authors:** Charlotte Grund, Martha M. Robbins, Catherine Hobaiter

**Affiliations:** 1 School of Psychology and Neuroscience, University of St Andrews, St Andrews, UK; 2 Department of Primate Behavior and Evolution, Max Planck Institute for Evolutionary Anthropology, Leipzig, Germany

**Keywords:** Gorilla communication, Language evolution, Great ape gesture, Gesture phylogeny, Intentionality, Signal

## Abstract

Over recent decades comprehensive catalogues of vocal, facial, and gestural signals have been established for most great ape species; however, a systematic description of wild gorilla gestural behaviour, particularly of the Eastern gorilla species, remains missing. We address this absence by cataloguing the physical form of gestural units used by 49 habituated wild mountain gorillas (*Gorilla beringei beringei*) from four social units in the Bwindi Impenetrable National Park, Uganda (*n* = 157 observation days over 8 months). We obtained a dataset of *n* = 3220 instances of intentional gesture, coded with a systematic ELAN-based framework (GesturalOrigins). Mountain gorillas employed a repertoire of 63 gesture actions, including potentially species-specific units, across 10 behavioural contexts. A latent class analysis on variants of gesture action expression split units further into 126 finer-grained forms (‘morphs’). We observed ~ 6 gestures per hour of observation time and species-level repertoire size was similar to those reported in both *Pan* species. Our study constitutes the first systematic description of the mountain gorilla gestural repertoire, providing a new understanding of their communication, filling current gaps in great ape gestural phylogeny, and complementing previous studies on their vocal signals. Living in cohesive, small-sized female-male bonded social units, gorillas show striking differences in social organisation as compared to *Pan* species and provide crucial context for theories on the potential ancestral states of human communicative behaviour.

**Supplementary Information:**

The online version contains supplementary material available at 10.1007/s10071-025-01977-8.

## Introduction

In their everyday social interactions non-human primates engage in sophisticated signal use that shares important commonalities with human linguistic communication. Some primate vocalisations, for example, seem to function referentially (e.g., Slocombe and Zuberbühler 2005; Cheney and Seyfarth 2018), while others show potential for syntactic or combinatorial structures (e.g., Crockford and Boesch 2005; Leroux et al. 2022). However, the clearest consistent evidence for *intentional* and *goal-directed* signal use—essential hallmarks of human linguistic behaviour—is found in primates’ gesturing during close-range (dyadic) social interaction (e.g., *macaques*: Canteloup et al. 2015; Gupta and Sinha 2019; *baboons*: Molesti et al. 2020; *mangabeys*: Schel et al. 2022; *spider monkeys*: Villa-Larenas et al. 2024; *siamangs*: Liebal et al. 2004a), with the largest and most flexibly deployed repertoires described in great apes (*chimpanzees*: Tomasello et al. 1985; Liebal et al. 2004b; Hobaiter and Byrne 2011a; Roberts et al. 2014; *bonobos*: Pika et al. 2005; Graham et al. 2017; western *gorillas*: Pika et al. 2003; Genty et al. 2009; *orangutans*: Bard 1992; Cartmill and Byrne 2007; Liebal et al. 2006; Knox et al. 2019). Gestures, defined as mechanically ineffective movements of body parts (or the body as a whole) employed in goal-directed communication to achieve specific social goals (e.g., Hobaiter and Byrne 2011a), at times combined with other signal types such as vocalisations (e.g., Wilke et al. 2017; Hobaiter et al. 2017) or facial expressions (e.g., Oña et al. 2019), are essential social tools with which great apes (and other primates) initiate, mediate, and terminate social interaction; that is, navigate their diverse social relationships.

The discovery of the ubiquitously intentional use of gestures in great apes placed them at the centre of the debate on candidate precursor systems for human language, with some researchers arguing for a predominantly gestural evolutionary origin (e.g., Arbib 2012; Corballis 2009; Hewes 1973). Current descriptions of great ape gestural repertoires suggest extensive overlap in gesture form across species (~ 50–80%), and greater overlap in more closely related species (Byrne et al. 2017). Furthermore, recent pilot studies revealed substantial use of the chimpanzee gestural repertoire by pre-verbal human children (Kersken et al. 2019) and an understanding of ape gestures in human adults (Graham and Hobaiter 2023), suggesting humans retain some access to a shared ape-typical system of bodily communication.

Over recent decades research efforts have increasingly included data on wild apes communicating in socio-ecological naturalistic contexts (e.g., *chimpanzees*: Hobaiter and Byrne 2011a; Roberts et al. 2012, 2014; Fröhlich et al. 2016, 2017; *bonobos*: Graham et al. 2017; *orangutans*: Knox et al. 2019); however, important pieces remained missing in the puzzle of naturalistic great ape gesturing, most notably from gorillas. While systematic descriptions of vocal repertoires in both gorilla species have been made (Harcourt et al. 1993; Salmi et al. 2013; Hedwig et al. 2014), gorilla gesture use has only been comprehensively studied in the western lowland gorilla subspecies in captivity (*Gorilla gorilla gorilla*: Genty et al. 2009; Pika et al. 2003; Tanner and Byrne 1999), where they showed particularly large gestural repertoires (Genty et al. 2009) when compared to similar studies of the two *Pan* species (both wild communities, cf. Byrne et al. 2017). It is unclear whether gorillas in the wild employ a similarly large variety of gestures in their every-day life, and to what ends. The captive context has been suggested to promote the use of a wider range of gesture types (Fröhlich et al. 2021), and the vast majority of gesturing observed in captive gorillas occurs during play (Genty et al. 2009), a particularly prolific behavioural context for great ape gestural expression, both in captivity (e.g., Genty et al. 2009; Liebal et al. 2004b; Pika et al. 2005) and in the wild (e.g., Fröhlich et al. 2016; Hobaiter and Byrne 2011b). However, while play tends to dominate gestural communication in captive apes, in naturalistic settings, non-play contexts also hold substantial significance for the use of gesture (e.g., Hobaiter and Byrne 2012).

The addition of gorilla gesture represents more than simply another species for the sake of phylogenetic completeness. We have increasing understanding of the importance of socio-ecological context on the expression and use of gesture (Fröhlich et al. 2021; Graham et al. 2022; Badihi et al. 2024), and in this respect gorillas represent a very different model system from the well-studied *Pan* species. The core of gorilla social organization is long-term male-female association, in which social units are typically comprised of at least one dominant adult male (silverback) and several adult females and their offspring (Harcourt and Stewart 2007; Yamagiwa et al. 2003). In contrast to the fission-fusion social organisation that is typical for the (larger) multi-male multi-female social units of *Pan* (Nishida 1968; Kano 1982; Gruber and Clay 2016), *Gorilla* species, in particular mountain gorillas, show a more cohesive grouping pattern (Harcourt and Stewart 2007; Young and Robbins 2023). Relatively consistent cohesion in gorillas may be promoted by females seeking protection from the silverback (their offspring’s father) to prevent infanticide by stranger males (Harcourt and Stewart 2007) and is likely to be supported by their comparatively small-sized groups and low food competition (Watts 1985; Grueter et al. 2016; Robbins and Robbins 2018). Living in high-altitude rainforests that typically show lower fruit tree density as compared to lower-altitude habitats, the mountain gorilla (*Gorilla beringei beringei*) is in general less frugivorous (Ostrofsky and Robbins 2020; Robbins et al. 2022) and shows greater social cohesion than the lowland gorilla—and than any of the other great ape species (Yamagiwa et al. 2003).

In both gorilla species, females may emigrate to neighbouring groups/silverbacks repeatedly across their lifetime (Stokes et al. 2003; Manguette et al. 2020; Robbins et al. 2009). However, within the genus *Gorilla* there is substantial flexibility in social structure and group composition, with about 40% of mountain gorilla groups being multi-male compared to the almost exclusive one-male system of western gorillas (Robbins and Robbins 2018). The flexibility in social structure across *Gorilla* species and the fluidity of social group membership within an individual’s lifetime stands in relatively stark contrast to the consistent formation of multi-male multi-female groups in *Pan* species, and where only the females emigrate from their natal group—and usually only once in their lifetime (Goodall 1986; Kano 1992).

The variation in species’ social organisation may have implications for interspecific variation in communication styles, with gorilla gesturing potentially being shaped by an increased need to flexibly adjust to new social settings. How gorillas communicate in their smaller-sized and cohesive, yet fluid and variable social system, structured by female-male bonding, provides crucial context for our understanding of how variation in social structure impacts the expression of ape gesturing (see: Graham et al. (2022) for an extended review on potential impacts of social structure on ape gesturing). Since early human social organisation may have shared core characteristics with gorilla social structure, for example in terms of social fluidity (Morrison et al. 2020), or the formation of family-like units based on female-male association, data on naturalistic gorilla gesturing are also crucial for theories on the ancestral state of human communicative behaviour (as are data on the two *Pan* species; Lameira and Call 2020). Gorillas also represent an important perspective on the influence of ecology on ape communication, for example in how a predominantly terrestrial versus arboreal lifestyle and associated differences in limb mobility may shape gestural expression, as seen in chimpanzees as compared to orangutans (Knox et al. 2019). As gorillas are the most terrestrial of all extant non-human great apes, next in line to the (almost exclusively) terrestrial humans, they constitute a particularly important point of comparison.

A systematic investigation into wild gorilla gesturing is thus needed to complement our understanding of natural gorilla-specific communication, to place gorillas within the larger picture of ape gestural phylogeny, and to explore how socio-ecological variability impacts great ape gesturing. As a start, we provide the first systematic description of the physical forms of mountain gorilla gestural signals. Adopting a component-based approach to repertoire construction (Grund et al. 2023), we first define the basic bodily movements (gesture actions) employed by individuals of all age classes in their natural social interactions, before we determine finer-grained sub-units of gesture action expression (‘morphs’) using a data-driven latent class analysis (LCA; see Mielke et al. 2024). We compliment our description of mountain gorilla gesture actions with an open access video catalogue (Grund et al. 2024), report individual repertoire sizes and gesturing rates based on age and sex, and estimate the contextual diversity of mountain gorilla gesturing by describing the behavioural contexts of signallers following each gestural interaction.

## Methods

### Study site and subjects

Research was conducted in the Bwindi Impenetrable National Park (BINP) in south-western Uganda (0°53′–1°08′N; 29°35′–29°50′E; McNeilage et al. 2001). The BINP comprises an area of approximately 331 km² of montane forest (BINP altitude range: 1160–2607 m above sea level; McNeilage et al. 2001) and is home to an estimated minimum population of 459 mountain gorillas (UWA Mountain Gorilla Census, 2019). C.G. collected data for 8.5 months (157 observation days) between 2019 and 2022 on four habituated social units (Mukiza, Rukara, Bitukura, Oruzogo) comprising individuals of all age classes (*n* = 50 individuals). Tables S1-S4 in the ESM list all Bwindi mountain gorilla subjects with information on their age, sex, and group association, and reports demographic changes between or during data collection periods (specified in Table S5).

### Video data collection

During observation hours (group follows) all social interactions (any behaviours with potential gesture use) were collected *ad libitum* using a handheld video camera (Panasonic HC-V770) set with 3-seconds prerecord. At times a second camera (GoPro Hero 7) was used to capture simultaneous social interactions. Each video clip was then coded for the individuals present, the predominant activities/contexts they were in, and whether there was gestural behaviour. The mountain gorilla video database contains *n* = 3543 video clips comprising ~ 220 h of video material collected during 553 h observation time and can be searched through the *Great Ape Video Ark* (Hobaiter et al. 2021).

Observation effort was balanced for the social units Bitukura and Mukiza. Rukara and Oruzogo were followed less often, largely due to differing visiting regulations during the Covid-19 pandemic (see Table S6 in the ESM for details on observation effort and data contribution per group).

### Gesture coding

From the video database C.G. coded all clips with gestural interaction data with the video annotation software ELAN (ELAN 2022) using the GesturalOrigins coding template (Grund et al. 2023; Figure S1 in ESM). The coding scheme and protocol along with definition sheets of all coded variables can be accessed at the following repository https://github.com/CharlotteGrund/Gestural_Origins_Coding-methods_paper).

We restricted our coding to those gestural signals that were *intentionally* produced, *socially directed* (towards a specific recipient), and employed with the communicative intention to receive a particular response, i.e., *goal-directed* (for more details on the definitions and implementation of these criteria see ESM section ‘Gesture coding’).

### Gesturing context

During the coding of gestural interactions, we annotate the behavioural context of signaller and recipient prior to and after each completed communication (following Grund et al. 2023). In order to describe the contextual diversity of mountain gorilla gesturing we compiled the behavioural context of the signaller at the end of each successful or unsuccessful gestural communication (*n* = 1462 communications, *n* = 49 signallers). For more details on the coded variable and context definitions see section ‘Signaller context’ and Table S8 in the ESM.

### Repertoire construction

We describe and categorise the physical form of gestural behaviour after the basic bodily movement (‘gesture action’) that the signaller performs. Here each gesture action is defined (prior to coding) on the basis of a ‘minimal action unit’ (MAU, Grund et al. 2023), the part of the gestural movement that sufficiently discriminates it from any other action type in the gestural system.

The initial definitions of ape gestural actions were compiled from a comprehensive comparison of great ape gesture types (Byrne et al. 2017), in which we retained only the movement type as the discriminating element (e.g., ‘hit’, ‘hitting’, ‘hit-foot’, ‘hit-hand’, ‘hit-two hands’ etc. were all assigned to the action ‘hit’). From this list of gesture actions, we further defined the MAU as the minimum section of the gestural movement required to recognise and discriminate between gesture actions—for example, this would include the section of the gestural movement required to extend a hand into a ‘reach’ but not any subsequent time or movement associated with holding it in place, moving the hand into a new gesture action, or recovering the hand to a resting position (for full details, see Grund et al. 2023).

We then separately code other descriptive aspects of the gestural behaviour (termed *modifiers*), for example: which body part the signaller used (e.g., hand vs. foot) or the use of rhythmic repetition (Hit vs. Hitting), per instance of gesture action use (i.e., per gesture *token*). The modifiers add further structural information about the specific expression of the gestural movement in an instance of use, and data-driven computational approaches can subsequently be used to detect consistent patterns in gesture action + modifier combinations (*morphs*) post-coding (see Figure S2 in ESM and section ‘Repertoire construction’ for more details). This approach differs from the use of fully pre-defined ethograms of gesture units, which include modifiers in the unit description from the outset (e.g., ‘arm raise’ or ‘feet hitting’).

We describe the gesture forms used by mountain gorillas at two levels: (1) the gesture action and (2) the morph repertoire. In the construction of the gesture action repertoire (1), we describe the basic gestural movements mountain gorillas employed, without considering other aspects of expression (e.g., which body part was used to perform the action). While we describe all gesture actions observed (even where *n* = 1), we only considered gesture actions that we observed at least three times as present in the species’ gesture action repertoire (cf., Hobaiter and Byrne 2011a; Graham et al. 2017). As the initial set of gesture actions were extracted from a comprehensive cross-species comparison—including data from *Pan* sp. (wild), *Gorilla gorilla* (captive), and *Pongo* sp. (captive; Byrne et al. 2017)—any basic gestural movement we observed that was not yet listed, was considered a ‘new’ (potentially mountain gorilla-specific) gesture action.

In the construction of more detailed gestural units, morphs (2), we applied the latent class analysis (LCA) approach developed by Mielke et al. (2024). We used the associated R package (‘wildminds’ version 1.0) to conduct statistical analyses. LCA is a statistical procedure that identifies subgroups (latent classes) in multivariate categorical data. In this case, the latent classes are based on the modifier levels within gesture actions and the resulting clusters are the morphs. In LCA it is assumed that each class is both mutually exclusive and exhaustive, i.e., each observation can only be assigned to a single class, with resulting morphs within each gesture action perfectly described by their specific configuration of modifier levels. The morph detection is, in itself, a flexible approach allowing greater or lesser splitting of the gestural units depending on the number and type of modifiers included in the analysis. In the present study we included four modifiers: *Body part*, *Contact recipient*, *Lateral use*, and *Repetition*. These are among the most well-represented modifiers employed in defining repertoires to date (e.g., Cartmill and Byrne 2007, 2010; Genty et al. 2009; Graham et al. 2017; Halina et al. 2013; Hobaiter and Byrne 2014, 2017; Leavens et al. 2010; Liebal et al. 2006; Pika et al. 2003; Pollick & de Waal 2007) and were relevant across the widest range of gesture actions in the dataset. Full definitions of the four modifiers are listed in Table S7 in the ESM, together with the level at which they were coded and analysed (as well as their relevance for different subsets of gesture actions). Finally, we only included gesture actions with at least 10 observations in the morph analysis (as there had to be some potential for variation in the data), with the threshold for inclusion as a morph set to a minimum of 5 observations (for more details on the procedure see Mielke et al. (2024).

After passing through the morph detection, a gesture action may be split into several morphs or may show just one typical expression. Thus, we differentiated *polymorphic gesture actions*, gesture actions (with > 10 observations) that were split into multiple morphs, from *unimorphic gesture actions*, gesture actions (with > 10 observations) that only ever occurred in one typical configuration of modifier levels (one morph). Finally, we described *unspecified* gesture actions, which were excluded from the morph detection due to too few cases in our current data to detect variation, i.e., gesture actions with < 10 observations (but over > 3).

### Comparison of ‘newly’ identified gesture actions with descriptions of bodily communication in the wider gorilla literature

The initial set of gesture actions in our coding framework was extracted from a comprehensive cross-species comparison of great ape gesture forms (Byrne et al. 2017) that included data from *Pan* sp. (wild), *Gorilla gorilla* (largely captive), and *Pongo* sp. (captive). We initially considered gesture actions that were ‘newly’ identified in the current study (i.e., not described in Byrne et al. (2017) to be potentially specific to mountain gorillas (see above). However, we recognised that some of these ‘new’ gesture actions might have already been reported in wider studies on gorilla communication or in other descriptions of their behaviour. To address this limitation, we assessed whether there was any evidence for each of the ‘potentially mountain gorilla specific’ gesture actions identified in our first step, to be present in the wider literature. We compared their definitions to existing detailed descriptions of captive western lowland gorilla gesturing (Genty et al. 2009; Pika et al. 2003; Tanner and Byrne 1999) as well as to other reports on gorilla bodily communication and estimated any cases of possible overlap of components of each ‘newly’ identified gesture action.

### Gesture datasets

The mountain gorilla gesture coding resulted in a dataset of *n* = 3220 gesture tokens and comprised data from individuals of all four social units, both sexes, and a wide range of ages (*n* = 49 signallers, *n* = 50 recipients, signaller ages ranged from 7 months to over 33 years (older ages are based on estimates); see Table S1-S4 in the ESM for more details). Only gesture actions with > 3 observations were included in the gesture action repertoire analysis, reducing the dataset to *n* = 3203 tokens. The latent class analysis was conducted on 94% of the original dataset (*n* = 3024 instances; all tokens with unclear modifier levels (*n* = 97) and gesture actions that had < 10 observations (*n* = 18 gesture actions; *n* = 99 tokens) were excluded). To estimate individual repertoire sizes and gesturing rates we only included data from the two social units with the most comparable datasets (Mukiza and Bitukura—for more details see section *Individual repertoire sizes and gesturing rate* below).

### Interrater reliability

To assess inter-rater reliability of the coding by C.G., C.H. re-coded a subset of gesture tokens (*n* = 170). Gesture tokens were selected randomly, but to a sample size that was proportional to each gesture action’s representation in the dataset and comprising ~ 5% of the entire gesture dataset). All variables analysed reached substantial (Cohen’s kappa: *k* = 0.64 with 83% percentage agreement: e.g., variable ‘*Directed_to’*) to perfect (*k* = 1.0: e.g., variable *Repetition_count*) levels of agreement (see ESM section ‘Interrater reliability’ and Table S9 for more details on how the test data were selected, the testing procedure, and the agreement level for each variable).

### Individual repertoire size and gesturing rate

To estimate individual repertoire sizes (IRS) and individual gesturing rates (IGR) across maturation and sex classes, we included data from the two social units for which we had comparable datasets (Bitukura and Mukiza; *n* = 2877 gesture tokens; *n* = 27 signallers; see Table S6 for more details on observation effort per group). As in previous studies (cf. Hobaiter and Byrne 2011a) we report individual repertoire size at two levels: (a) *IRS_1* = the total number of distinct gesture actions employed by the individual (even where *n* = 1) and (b) *IRS_3* = the total number of distinct gesture actions that were used at least three times/signaller.

We estimate individual gesturing rate by calculating (a) *IGR_obs* = the number of gesture tokens the individual produced/h observation time (social unit follow), and (b) *IGR_vid* = the number of gesture tokens the individual produced/h of video footage in which the individual was recorded.

### Categorisation of sex and maturation classes

We classified Bitukura and Mukiza individuals into four broad maturation categories (Mat. cat):

*Mat. Cat-1*: *infants* (0–3 years, both sexes); *Mat. Cat-2*: *juveniles* (> 3–6 years, both sexes); *Mat. Cat-3*: *subadults* (> 6–8 years, both sexes) *+ young nulliparous females* (females 8 > 12 years without infants) *+ blackbacks* (males: > 8–12 years); *Mat. Cat-4*: *adults* (males > 12 years; parous females; adult nulliparous females > 12 years). This categorisation closely followed Breuer et al.’s (2009) classification of gorilla maturation based on age for the infant, juvenile, and subadult subcategories (both sexes) as well as the blackback and adult male subcategories. We decided to categorise fully matured (adult) females not by age alone but to include markers of behavioural maturation (here parity), since the age at first birth varies (Robbins et al. 2023), but may be a more suitable indicator of adult female maturation for our study (because of the likely impact of social maturity and motherhood on gesturing). While the categorisation into young nulliparous and adult parous females was straightforward, one adult female (Kamuga) in the Bitukura group to our knowledge never gave birth but was clearly an adult (> 20 years). We included her in the adult female category despite being potentially nulliparous. Note that due to the specific demographics of the social units during the study period (all new-born offspring were male; cf. ESM, Table S1-S2), the infant category is biased towards males.

As the study period covered three years and was split into four data collection seasons that lasted for 1–3 months, some individuals contributed data to more than one maturation class. Having hard cut-offs for individual maturation classes within the same season seemed unreasonable, in particular when calculating signal repertoire sizes – for the analysis we therefore made sure that the same individual was only represented in a single class within each season. For the maturation categories based on age alone (infants, juveniles, subadults, blackbacks, adult males) we proceeded as follows: whenever an individual changed maturation class within a data collection season, we allocated gesture tokens to the class that was most well represented in their data that season. Whenever a female gave birth within a season for the first time, we categorised her as parous even if she gave birth towards the end (and not as nulliparous – note that this was only the case for one individual (Bwebisha)).

### Data analyses and visualisation

All analyses were conducted in the statistical programming software R version 4.3.0 (2023-04-21). Code, including R markdown documents can be accessed on GitHub (https://github.com/CharlotteGrund/mg_repertoire).

## Results

### Mountain gorilla gesture action repertoire

Mountain gorillas used 63 distinct gesture actions to communicate with each other (for definitions see Table 1), and we recorded an average 6 gesture tokens/h of daily observation time (range: 0.3–29.8). Within the observed gesture actions, *n* = 15 were newly identified (with the potential to be mountain gorilla-specific); thus, approximately 76% (*n* = 48/63) of mountain gorillas’ basic gesture actions were shared with at least one other great ape species. *N* = 12 gesture actions were observed on fewer than three occasions and are not currently incorporated into the mountain gorilla repertoire (gesture actions in italics in Table 1). *N* = 21 gesture actions reported in other ape species were not observed in Bwindi mountain gorillas (gesture actions struck out in Table 1). Table S11 in the ESM lists all great ape gesture actions in alphabetical order, indicating their presence or absence in the mountain gorillas.

**Table 1:** Definitions of great ape gesture actions together with newly identified mountain gorilla units. The repertoire of great ape gesture actions is taken from Grund et al. (2023), based on systematic studies in the existing literature (e.g., Byrne et al. 2017), and combined with the new gesture actions described in this study (newly identified gesture actions are indicated with asterisks). Gesture actions considered as part of the mountain gorilla repertoire (observed on at least three occasions) are in bold and listed in alphabetical order in the upper part of the table. Gesture actions that were observed on fewer than three occasions are in italic font in the middle part of the table. Gesture actions seen in at least one other ape species but not seen in mountain gorillas are indicated as struck out in the last section of the table. Italic font in the gesture definition column indicates additional information not necessarily part of the gesture action definition (comments relating to categorisation). More detailed definitions of newly identified gesture actions, including MAU start and stops are presented in Table S10. For MAU definitions of all other gesture actions listed see Grund et al. (2023)

Gesture action	Gesture definition
**Beckon**	A scooping movement from one or more of the joints (e.g., fingers, wrist, elbow), movement extends towards recipient and then back to the signaller in one active motion.
**Bite**	Signaller's mouth/teeth close on the recipient’s body - this may be very brief or could be held in place.
**Bite: threat**	Signaller opens mouth rapidly as if preparing for a bite (often with a movement towards recipient) but moves past or away quickly before making contact.
Body cross*	Signaller is holding arm across their own body (i.e., if the right arm is used it is held across to the left side of the body), either in the air or clutched to the signaller’s chest/front.
Bump into*	Signaller approaches (running or slowly walking) recipient and bumps into recipient’s body (full body movement). It is different from “Lean in” as the action is a fast (dynamic) movement and the contact with the recipient very short.
**Chest beat**	Signaller’s hands make (alternating) short audible contact with signaller’s chest (always produced while bipedal, rhythmic, long-distance audible chest beat).
**Chest beat informal**	Signaller’s hands make (alternating) short audible contact with signaller’s chest (rhythm or beats can be irregular, slower, or less formal etc.). Produced bipedally or sitting, lying etc. *Has to include at least 3 hits, otherwise treated as ‘Hit Self’. In Byrne et al. 2017 no distinction made between Chest beat and Chest beat Informal (but see ‘Body drum’).*
**Dangle**	Signaller hangs from one or both hands (or feet) from a branch with at least one limb not in contact with branch, some movement of body as a result of hanging. Includes ‘Dangle with shake’ where the signaller produces an additional shake of arm(s) or leg(s). *Dangle was not coded as often as a gesture action because of difficulties to discern it from play actions. It also only ever was performed by immatures, and almost exclusively to initiate play.*
**Embrace**	Signaller wraps one or both arms/legs around recipient (not necessarily only affiliative).
**Fling**	Rapid movement of hand (from wrist) or arm (from elbow or shoulder; rarely with foot or head) away from the signaller’s body, typically towards recipient. Energy of stroke is focused on way out away from signaller, more relaxed on way back.
Gaze stance*	Signaller positions body and head towards the recipient’s face, stares at them and ‘holds’ their gaze. The intensive ‘looking’ lasts at least 2 seconds or longer (unlike e.g., a quick attention checking). The gaze is accompanied by a stiff (motionless) body posture (standing or crouching in front of the recipient), and so is different from the Gaze hold. *See discussion.*
**Grab**	The hand or foot is firmly closed over part of the recipient’s body or a handful of hair.
**Grab hold**	Same as "Grab" but hand(s)/foot of signaller stays closed around recipient’s body for > 2 seconds.
Head avert*	Signaller’s body is positioned towards the recipient’s body but the head is tilted away from the recipient in an exaggerated almost 90 degree angle. May be interrupted by short monitoring of the recipient’s (re)action (at times this attention checking can resemble the description of a (horizontal) head shake but the movements in the head avert are stiff, whereas the head shake is a relaxed throwing the head in different directions)
**Head stand**	Signaller bends forward and places head on or very near to ground and pauses, at least briefly, in this position. Contact may be made with recipient’s body.
Hit fake*	The signaller is positioned close to the recipient and both individuals are stationary. The signaller raises the arm/hand and lashes out as if to hit the recipient but does not make contact. The outward and backward movement have the same speed and often the arm/hand is still held up after the first ‘faked’ hit for a while (and the gesture action is then either repeated or the arm slowly put back into a neutral position). If contact is made this gesture action is classified as Hit tap.
Hit non-recipient*	Signaller approaches/positions towards recipient with a young offspring (or a ‘stolen’ infant) clinging to the signaller’s front (belly/chest) and (very) softly hits on the infants back similar to a ‘chest beat’ (so alternating rhythmical hand movements on the clinging infant) while gaze and body posture clearly indicate that the behaviour is directed at the other individual (recipient). Note that very soft (rhythmical) hitting on young infants is also observed in mother-offspring interaction.
**Hit object/ground**	Signaller makes a short hard contact with the ground/object; energy in movement is focused on the way out, contact can be brief or remain in place for longer. Typically occurs in front of or to the side of the signaller’s body (rather than straight up and down as in Stomp). Produced with a variety of body parts - typically hand, fingers, knuckles, fist, foot.
**Hit object/ground with object**	An object is brought into short hard contact with another object or the ground.
**Hit recipient**	See “Hit object/ground”, but signaller makes deliberate contact with the recipient as part of action.
**Hit recipient (soft)**	See “Hit object/ground (soft)” but contact is deliberately made with the recipient’s body. *There is clear variation in hit intensity for the gesture action ‘Hit recipient’ in the mountain gorillas. This variation was not observed in the two other very common ‘hit’ gesture actions (hit self and hit object ground).*
**Hit self**	See “Hit object/ground”, but signaller makes deliberate contact with own body as part of action.
Hit tap*	The signaller is positioned close to the recipient and both individuals are stationary. The signaller raises the hand, lashes out and makes (very) short contact with the recipient’s body. The outward and backward movement have the same speed and often the arm/hand is still held up after the first ‘tap’ for a while (and the gesture action is then either repeated or the arm slowly put back into a neutral position). In movement form similar to ‘Hit fake’ but there is contact made.
Jab*	Signaller makes a short hard contact with the recipient’s body, typically with foot (occasionally with fist), unlike in “Hit recipient” the energy is rapid on both the way to and from the recipient.
**Jump**	While bipedal both feet leave the ground simultaneously, while quadrupedal all four limbs free of ground simultaneously; accompanied by horizontal displacement.
**Kiss**	Gentle contact with the mouth that doesn't hold the recipient’s body (see "Bite"). Includes the ‘share air’ kiss type where they breath on each other while almost touching.
Lay on*	The signaller lifts their leg to the side (like a dog wanting to urinate) and lays it on the recipient’s body. Depending on the size difference between signaller and recipient this can look quite acrobatic.
Lean in*	The signaller approaches the recipient and leans into the recipient’s body (usually the signaller’s shoulder or head to the recipient’s chest). It is a contact gesture but if it happens during locomotion, contact may not be stable throughout the duration of the gesture.
**Locomote bipedal**	Signaller stands bipedally and takes at least one step with each foot.
**Locomote gallop**	An exaggerated running movement where the limbs movements are typically stiff (straightened) and rhythmic.
Locomote recipient*	Signaller finishes a locomote gesture (bipedal, gallop, stiff walk) by moving over the body of the recipient; includes some contact.
**Locomote stiff run**	An exaggerated walking movement with stiff limbs and forelimbs held in a straightened position (running)
**Locomote stiff walk**	An exaggerated walking movement with stiff limbs and forelimbs held in a straightened position (slow locomotion)
**Lunge**	Signaller’s body is rapidly thrust towards recipient. No contact is made.
Object drop(*)	The signaller takes an object (e.g., a stick or moss) and drops it close to (or onto) the recipient. *Potentially the same gesture action as leaf drop in bonobos – not included as new*
**Object in mouth**	Signaller holds an object (e.g., a small branch) in mouth. Hands should not normally be involved. *In the mountain gorillas this includes attached objects.*
**Object move**	Object is displaced, contact is maintained through movement. While movement may not be in a single direction it does not include the rapid back and forth of ‘Object move: shake’. Object is free to move and not attached to environment (e.g., a fallen branch). *Note that in rare cases a third-party may be used as the ‘object’ - communication should still be clearly directed to the recipient (so this should not be a triadic interaction).*
Object move: fiddle*	Signaller grabs or touches vegetation in front of the recipient and ‘fiddles’ with it. Object (usually the leaves of a sapling, or the sapling itself) movements look uncoordinated (i.e., going into various directions with no clear pattern) but are calm and slow. Movements usually come out of the hand/wrist or fingers.
**Object move: shake**	Repeated back and forth movement of an object (typically one still attached in the environment, e.g., sapling). Object movement must be controlled by the signaller’s hand/foot actively shaking the object and not a biproduct of the flexibility of the object.
Object stance*	Signaller grabs a tree trunk or liana usually above shoulder height and leans into it (almost as if stretching). The body is stiff and the posture held in place while there is only little movement of the object that supports it (if any at all, unlike object move).
**Over stance**	Signaller pauses while standing with at least one limb that has been moved into position and held over the recipient’s body. In full form signaller’s body forms a bridge over the recipient.
Pivot(*)	Signaller grabs an object (e.g., a small tree trunk or a firm branch) and swings his body in a semicircle around the object’s axis.
Place on object/ground*	Arm is extended in front of the body and hand (or fingers, knuckles) placed on an object or the ground in a location between the signaller and recipient (its usually the ground, but there may be thick vegetation/ or a log in that place). The movement of the arm/hand towards the object/ground is coordinated, the contact may be relatively short or last longer than 2 seconds. *Note that this is not the same gesture action as Touch object, a gesture action that is reported for chimpanzees and bonobos in the context of food sharing (where signallers touch the desired object).*
**Present**	Body or body part moved to deliberately expose an area to the recipient’s attention (excludes present fingers/hand/toes/foot in a ‘reach’ form). Contact is sometimes made during this gesture.
**Pull**	Same as “Push” but the force is exerted away from the recipient’s body (not effective in achieving the goal). Usually involves a “Grab” from the hand/foot that is not coded as a separate gesture. “Pull” vs. “Pull (directed)”: the direction of the movement afterwards is not consistent with the direction of the pull.
**Push**	Contact with recipient’s body (typically hand or foot) and the force is exerted into the recipient’s body (not effective in achieving the goal). “Push” vs. “Push (directed)”: the direction of the movement afterwards is not consistent with the direction of the push.
**Raise**	Raise body part (typically hand or arm) in a generally vertical movement, often with a brief pause near the top of the movement.
**Rake object/ground**	Signaller holds hands with fingers stiff and spread in order to displace objects towards their body, e.g., dry leaves. *In the mountain gorillas performed before hit ground.*
**Reach**	Body part (typically arm/hand) extended towards the recipient (or in their direction) with no contact.
**Roll over**	Signaller rolls or rocks so that their back is on the object/ground exposing their stomach and holds position.
**Rub**	Signaller pushes/rubs body part up and down against body of recipient (typically with hands or genitals).
**Shake**	Signaller moves part of their body quickly and repeatedly back and forth (typically hand/arm or head). Movement is loose and typical form has several repetitions.
**Spin: pirouette**	Signaller stands and turns around their body’s vertical axis while (at times) also displacing along the ground. All spin gesture actions need to include a full 360 degrees of turn (movement is more than would be required for locomotion).
**Spin: side roulade**	Signaller is lying down and turns around their body’s vertical axis while also displacing along the ground. All spin gesture actions need to include a full 360 degrees of turn (movement is more than would be required for locomotion).
**Stance bipedal**	Signaller moves into bipedal stance and holds position (must not be in order to achieve other action, e.g., standing to see something arriving). *Observed over 3 times in this study; however, only few cases and only immatures – unless more data in the future, maybe exclude.*
**Stiff stance**	Signaller holds stiff quadrupedal body position with stiff limbs and forelimbs (often accompanied by ‘tight lips’ facial expression).
**Stomp object/ground**	The foot (or sometimes hand) is lifted vertically and brought into short hard contact with the ground (or object). Usually this is the surface that the signaller is standing/sitting on. Typically, with sole of foot/flat or hand/fist.
**Stroke**	Active gentle movements of the signaller’s palm and/or fingers (rarely other) on the recipient’s body. May include movements in more than one direction.
**Swing**	Smooth continuous motion of body part (normally arm of leg) back and forth (may be repeated).
**Throw object**	Object is moved and released so that there is displacement of the object through the air after moment of release.
**Touch**	Light contact (typically of the fingers, knuckles, hand, or foot, rarely other) on the body of the recipient, contact < 2 seconds.
**Touch long**	Same as ‘Touch’ but contact is held for 2 seconds or longer. *Termed ‘Hand(s) On’ in Byrne et al. (2017). In some studies, ‘Hands on’ is only referring to touches on the head (e.g., Genty et al. 2009; Pika et al. 2003; Luef & Liebal 2013)*
Turn*	Signaller gazes back at the recipient and then makes an exaggerated half-turn with his/her body and head (one fore- and/or hindlimb may be raised during the turn). Similar to Beckon, however unlike Beckon, the movement is around the signallers own body axis.
*Big loud scratch*	Loud exaggerated scratching movement on signaller’s own body (must not be followed by self-grooming). A single BLS might include a small change in movement angle; but if contact stops or body part changes code as new BLS. *There is one possible occasion of the use of a big loud scratch gesture action in the mountain gorillas, but the intentional use was not clear.*
*Bounce*	Rhythmic vertical up-down movement (or’bobbing’) of the signaller’s body – in chimpanzees all hands and feet remain on the ground (rarely hands may be free of ground). *There is one clear example of a bouncing movement in the mountain gorillas seen performed bipedally before a chest beat (adult female).*
*Bow*	Signaller bends forward from the waist while standing bipedally. Note that head should not make contact with ground, and in typical form body is held straight and only lowered a little (compare: Head stand). *There is one example of a bow movement in the mountain gorillas seen performed by an immature individual.*
*Finger(s) in mouth*	Signaller inserts finger(s) - usually palm down - in the mouth of recipient with contact. Signaller makes movement into recipient's mouth - recipient might open or close mouth a little, but not the same as actively moving towards the other individual's hand to bite it. *There is only one example of this gesture action in the mountain gorillas.*
Freeze*	Signaller is approached by the recipient (e.g., the signaller is positioned in the travel path of the recipient) and instead of e.g., moving away or continuing with their activity the signaller ‘freezes’ into a rather unusual posture (e.g., crouching on the belly exposing the back or lying on the back with limbs stretched to the side exposing the belly).
*Hit recipient with object*	An object is brought into short hard contact with the body of the recipient. *There are two relatively clear observations in this study.*
Individual move*	Signaller grabs an individual and drags it along the ground as if it was an object. If this was directed at the individual being dragged and not effective in achieving the goal, the action was coded as Pull. However, there were a few occasions where the signaller seemingly just ‘used’ the individual he/she dragged as an object, while directing gaze and other behavioural markers at a different individual. *As this behaviour was only observed a few times and generally difficult to analyse we did not include it in the repertoire.*
Jab at*	See Jab - same action but signaller deliberately avoids making contact with the recipient's body.
Object clamp*	Signaller takes an object and carries it by clamping it between body parts. *There is only one example (here the signaller clamped a stick between the head and his chest).*
*Object on head*	Signaller places detached object on their head and leaves it in place. Hand may remain in contact with object for balance. *In mountain gorillas one occasion of this gesture action was observed but the object was attached.*
*Spin: somersault*	Signaller’s body is curled into a compact position on the ground and rolled forward or backward so the feet are brought over the head and returned to sitting position. All spin gesture actions need to include a full 360 degrees of turn (movement is more than would be required for locomotion). *Behaviour generally present but spin somersault was only used twice as a potentially communicative signal.*
*Water splash*	Signaller's limb (typically hand) is moved vigorously through the water so that there is audible displacement of the water. *Bwindi mountain gorilla immatures often play with water/mud, however only observed once as a gesture action in a play invitation.*
Butt	Signaller's head is briefly and firmly pushed into the body of the recipient (contact is typically short).
Circle	Signaller rotates hands around each other (in gorillas like the start of a chest beat but without any contact with chest). *Circling movements are often observed in the build-up phase of (usually immature) chest beats (so before the actual contact hits, maybe as a sort of ‘finding the rhythm’). “Circle” has not been observed as a separate gesture action in this study. In Byrne et al. (2017) called “Disco arms shake” (includes “Circle hands”)*
Clap	Signaller moves both palms towards each other which are brought together with audible contact (may be repeated).
Crouch	Signaller lowers body by bending knees and/or elbows, while maintaining at least three points of contact with ground. *Not observed in this study but see the gesture action Freeze.*
Flap	Flap movement of extremities, typically with one or both legs. Signaller sits with knees bent and opens/ closes one or both legs to side (can be repeated).
Gaze hold(*)	Signaller looks towards the recipient's face and'holds' their gaze (moving their head if needed as the recipient moves theirs), holds for > 2 seconds. *Termed ‘Look’ in Byrne et al. (2017). Mountain gorillas employ a gesture action that would fit the label ‘Gaze hold’; however, in mountain gorillas the gaze (which is more an intense starring) is accompanied by a stiff body posture and other behavioural markers. It is a very distinctive behaviour and does not resemble ‘peering’ (or ‘Look’). We therefore classified it under the gesture action ‘Gaze stance’. While it is reported, there was not enough example data for ‘Gaze hold’ available from other species for a thorough comparison.*
Hit object/ground (soft)	See “Hit object/ground” but contact is gentle (as in tap for example). *No obvious variation in hit intensity observed in the mountain gorillas.*
Hit self (soft)	See Hit soft object/ground but contact is deliberately made with signaller's body. *No obvious variation in hit intensity observed in the mountain gorillas..*
Knock object	Signaller's knuckles (or rarely heel) brought into short hard audible contact with an object like a tree buttress to make a clear sound.
Leaf clipping	Signaller tears strips from leaf (or leaves), or across leaves, held with the hand/teeth using the teeth or hands to tear; produces conspicuous sound.
Leaf clipping: leaf drop	Leaf or leaves are plucked off stem with signaller's fingers or mouth and quietly dropped. May be pulled/torn at petiole. Note that there is little tearing action on the leaves as seen in leaf clipping. *Not observed in this study but see ‘Object drop’ gesture action.*
Out	Typically, the signaller's arm is extended out from the shoulder to the side of the body, elbow and hand are held in line.
Poke	Short firm contact made with the signaller’s fingers held straight and ‘pushed’ briefly into the recipient’s body. *Could be confused with ‘Hit tap’*
Rake self	Signaller holds hands in distinctive position with fingers stiff and spread and pulls them over their body (e.g., over head, may be repeated; coded as a single gesture unless a clear break/change in movement). Rarely seen - often in association with temper'tantrums'.
Rocking: bipedal	Signaller moves body back and forth or side to side while standing bipedally.
Rocking: sitting	Signaller moves body back and forth, or side to side, while sitting (includes single/half'Rock' movement where the signaller moves back and holds).
Stomp recipient	See ‘Stomp object/ground’ but contact is deliberately made on recipient's body. Note that this typically occurs only when standing/sitting on top of someone, otherwise consider if Hit recipient a better fit. *Not observed.*
Throw threat	Object is lifted into position to throw it but is held in that position (typically raised above shoulder) without release. *There is one possible observation of a throw threat in the mountain gorillas, while it was a communicative behaviour, it was unclear whether the initial plan was to throw the object.*
Thrust	Groin is pushed forward towards recipient (may be repeated).
Touch object	Same as ‘Touch’ but contact is made with a specific object, usually food (begging/food sharing context). *See comment for ‘Place on object/ground’.*
Wave	Large loose back and forth movements of the signaller's arms while raised above the shoulder.

**Fig. 1:**
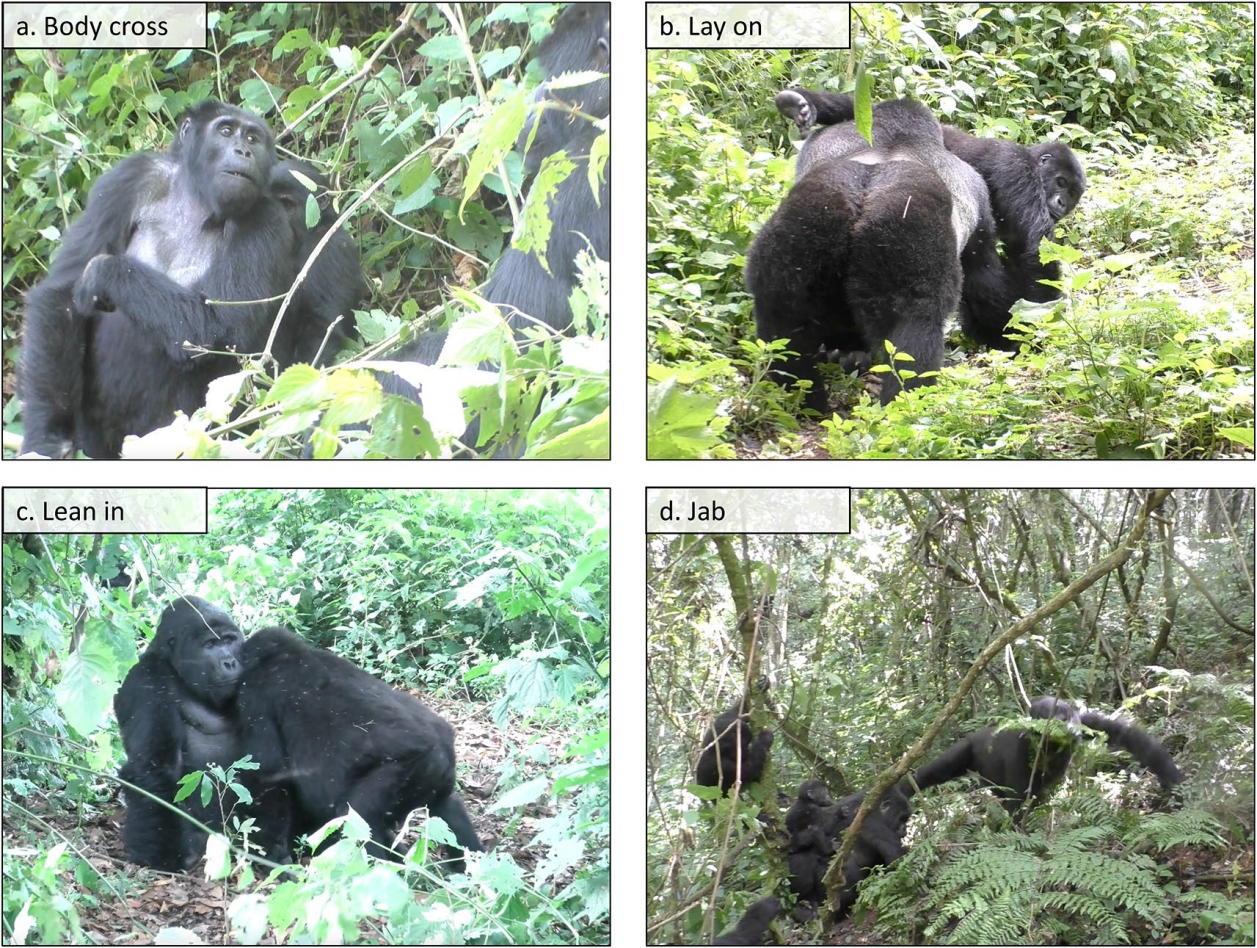
Potentially mountain gorilla specific gesture action examples. There are 15 gesture actions in the repertoire that have not been previously described in the great ape gesture literature and have the potential to be mountain gorilla specific. Four are illustrated here: *Body cross* (**a**), *Lay on* (**b**), *Lean in* (**c**), and *Jab* (**d**). The pictures capture the point in time where the gesture action is fully in place (MAU end, see Table S10 in ESM)

Video examples of all described mountain gorilla gesture actions (including those observed < 3 times) are available online (10.5281/zenodo.13341597). Figure 1 shows example images of four of the newly identified gesture actions, *Body cross* (a), *Lay on* (b), *Lean in* (c), and *Jab* (d).

**Fig. 2:**
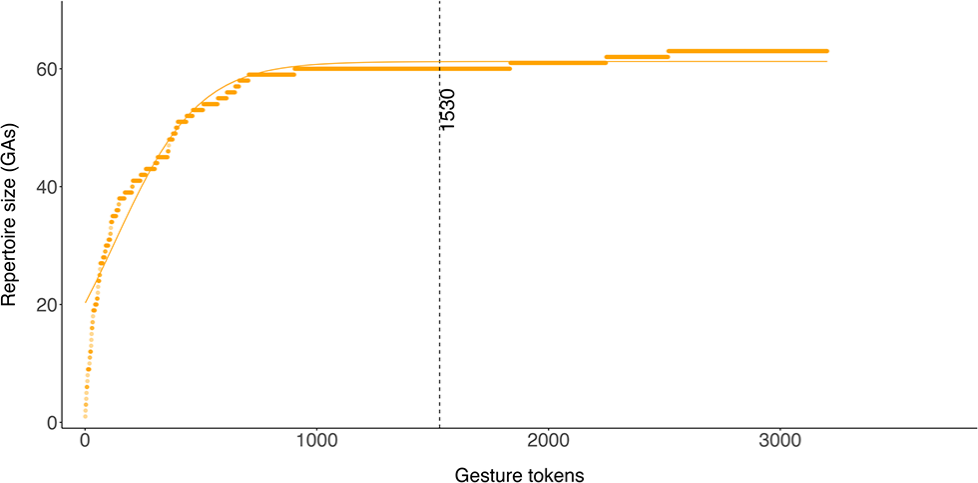
Detection asymptote for gesture actions. When plotting the identified repertoire units (here gesture actions) against the number of observations (gesture tokens), visual assessment of the asymptote allows us to estimate how likely it is that we have been able to fully describe all (habitual) gestural behaviour. We included all gesture actions (GAs) with at least 3 observations (*n* = 63 gesture actions; *n* = 3203 gesture tokens)

The plot of mountain gorilla gesture action repertoire size against the number of gesture tokens appears to reach asymptote at approximately *n* = 1530 (or 48%) of the total dataset (Fig. 2) suggesting that we have been able to catalogue the gesture actions habitually used by Bwindi mountain gorillas, and that while additional gesture actions may be identified in the future (for example, from among the gesture actions seen < 3 times, Table 1), further gesture actions are likely to be rare or only used infrequently in this population.

The ‘repertoire of regular use’ (~ 70% of gesture tokens, with gesture actions ordered from most to least frequent) included 16 gesture actions (*Push*, *Present*, *Grab*, *Touch*, *Object move*, *Hit object*, *Chest beat informal*, *Pull*, *Gaze stance*, *Reach*,* Stiff stance*, *Hit recipient*, *Locomote stiff walk*, *Embrace*, *Grab hold* and *Touch long*). Around half of the gesture actions observed (*n* = 32) contributed less than 1% of use. While the percentage representation of potentially species-specific gesture actions in the mountain gorilla repertoire was relatively high (15/63; 24%), their use contributed only 11% of tokens to the dataset (352/3203). The four most commonly employed newly identified gesture actions were *Gaze stance* (36% of all species-specific gesture tokens), *Lean in* (11%), *Place on object* (9%), and *Body cross* (7%).

The average gesture action would have been observed on 51 occasions if gesture action selection from within the repertoire was random (*n* = 63 gesture actions, *n* = 3203 tokens). The deviations from this mean value (Dev. average contribution) listed in Table 2 indicate to what extent each gesture action was under- or over-used, relative to a baseline of random signal selection.

**Table 2:** Number of tokens per gesture action and contribution to the dataset. The table lists gesture actions according to their token contribution (%) from the most frequent to the least frequent gesture actions. Dev. Average contribution = deviation from the Average contributed tokens (51 tokens per gesture action). Cumulative contribution (%) = summed percentages of token contribution to the data (in order). * potentially mountain gorilla specific gesture action

Gesture action	Token contribution (%)	Dev. average contribution	Cumulative contribution (%)
*Push*	8.6	223	8.6
*Present*	6.5	155	15.1
*Grab*	6.2	146	21.3
*Touch*	6.0	140	27.3
*Object move*	5.0	109	32.2
*Hit object*	4.8	101	37.0
*Chest beat informal*	4.5	92	41.5
*Pull*	3.9	74	45.4
Gaze stance*	3.9	73	49.3
*Reach*	3.8	71	53.1
*Stiff stance*	3.6	63	56.7
*Hit recipient*	3.5	61	60.2
*Locomote stiff walk*	2.6	31	62.8
*Embrace*	2.6	30	65.4
*Grab hold*	2.3	23	67.7
*Touch long*	2.2	20	69.9
*Raise*	1.9	8	71.8
*Bite threat*	1.4	-6	73.2
*Hit recipient soft*	1.4	-6	74.6
*Hit self*	1.4	-7	76.0
*Kiss*	1.3	-9	77.3
*Locomote stiff run*	1.3	-11	78.6
*Chest beat*	1.2	-12	79.8
*Bite*	1.2	-13	81.0
*Fling*	1.2	-14	82.2
Lean in*	1.2	-15	83.4
*Object shake*	1.2	-15	84.6
*Stroke*	1.1	-18	85.7
*Stomp object*	1.0	-19	86.7
Place on object*	1.0	-20	87.7
*All remaining gesture actions with <1% token contribution to the repertoire (in order)*: Spin pirouette, Beckon, Body cross*, Locomote recipient*, Bump into*, Lunge, Locomote gallop, Over stance, Jab*, Swing, Object stance*, Hit tap*, Dangle, Shake, Pivot, Lay on*, Head stand, Hit fake*, Rake object, Head avert*, Hit non-recipient*, Locomote bipedal, Rub, Spin roulade, Turn*, Hit object object, Object drop, Object mouth, Roll over, Stance bipedal, Throw object, Jump.			

### Variation in gesture action expression

Of the 45/63 gesture actions that showed sufficient cases to be included in the latent class analysis (LCA), *n* = 19 were *unimorphic* (i.e., only occurred in one typical configuration of modifier levels; 30% of the repertoire, and 30% of the gesture tokens in the LCA dataset), and *n* = 26 were *polymorphic* (i.e., showed at least two consistent variants of expression; 41% of the repertoire, 66% of gesture tokens in the LCA dataset), with the number of morphs per polymorphic gesture action ranging from 2 to 8 (mean = 3.4, sd = 1.5). 4% of gesture tokens included in the LCA could not be assigned to a particular morph at this time.

**Fig. 3:**
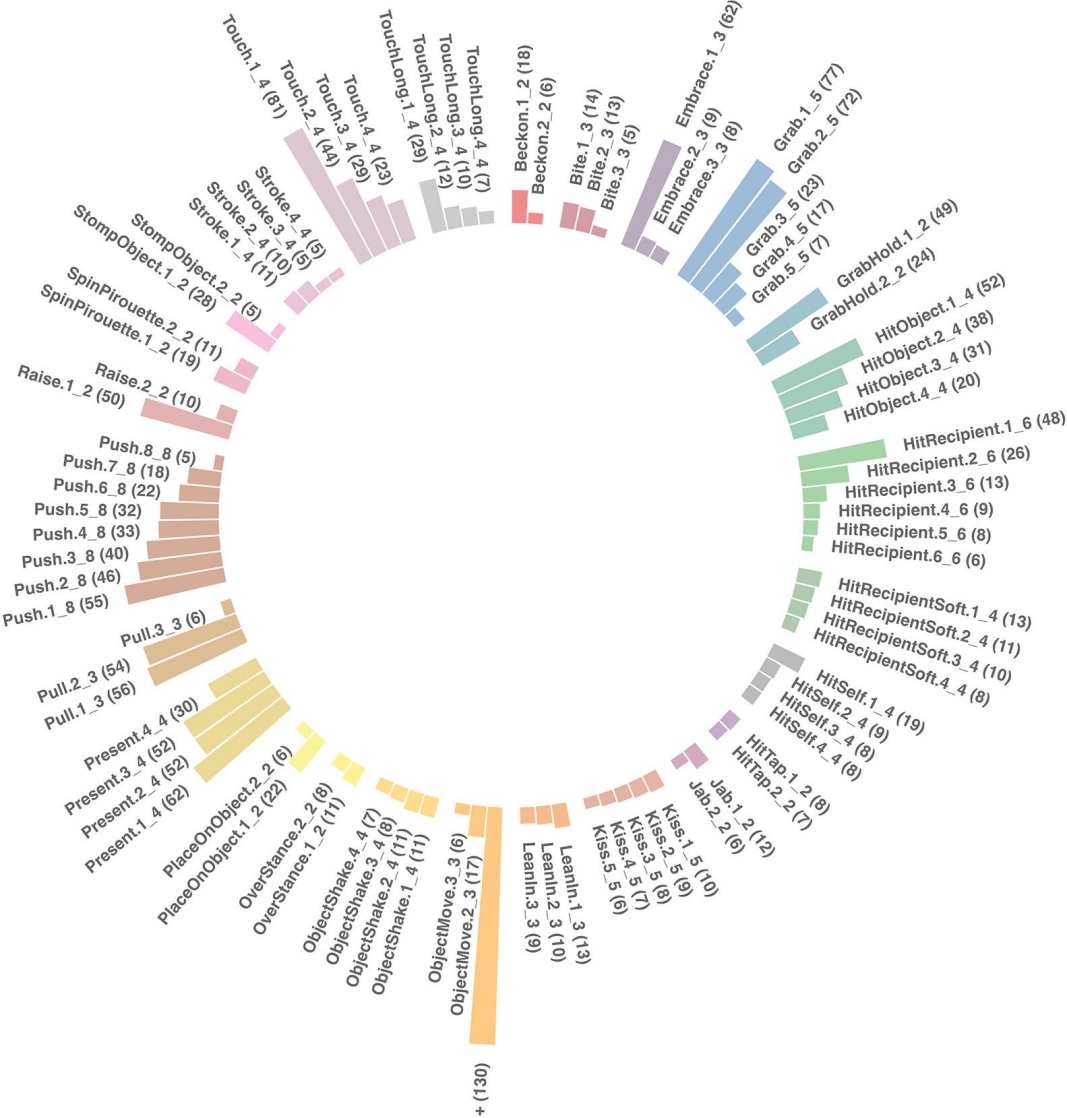
Token distribution of mountain gorilla gesture morphs in polymorphic gesture actions. The mountain gorilla morph analysis resulted in 126 gesture morphs. Only the morphs of polymorphic gesture actions are represented here (*n* = 89 morphs, *n* = 26 gesture actions). Morphs belonging to the same gesture action are grouped next to each other and are indicated with the same colour. The suffix ‘1_3’ indicates the first morph of a gesture action that has three morphs in total, and so forth. For clarity we removed the label of the first morph of *Object move* (ObjectMove.1_3) and replaced it with +

In total, the morph analysis resulted in 126 morphs (*n* = 19 unimorphic gesture actions; *n* = 89 morphs of polymorphic actions; *n* = 18 unspecified morphs of gesture actions with at least 3 observations but with too few cases for inclusion in the LCA). Morph detection asymptote was reached at ~ 1395 tokens (Figure S3 in ESM). The modifier configurations that define each morph are described in Table S12 in the ESM. Figure 3 shows the token distribution in the *n* = 89 morphs within the polymorphic gesture actions.

With morphs as the unit in the repertoire, the ‘repertoire of regular use’ comprised *n* = 37 gesture units. Of these 37, the 10 most commonly used gesture morphs were *Chest beat informal*.*1_1*, *Object move.1_3*,* Gaze stance.1_1*,* Reach.1_1. Stiff stance.1_1*,* Locomote stiff walk.1_1*,* Touch.1_4*,* Grab.1_5*,* Grab.2_5*, and *Embrace.1_*3. Table S13 contrasts the most frequently used gesture units when considering gesture actions vs. morphs as the unit in the gestural repertoire.

### Gesturing rate and repertoire unit use

**Fig. 4:**
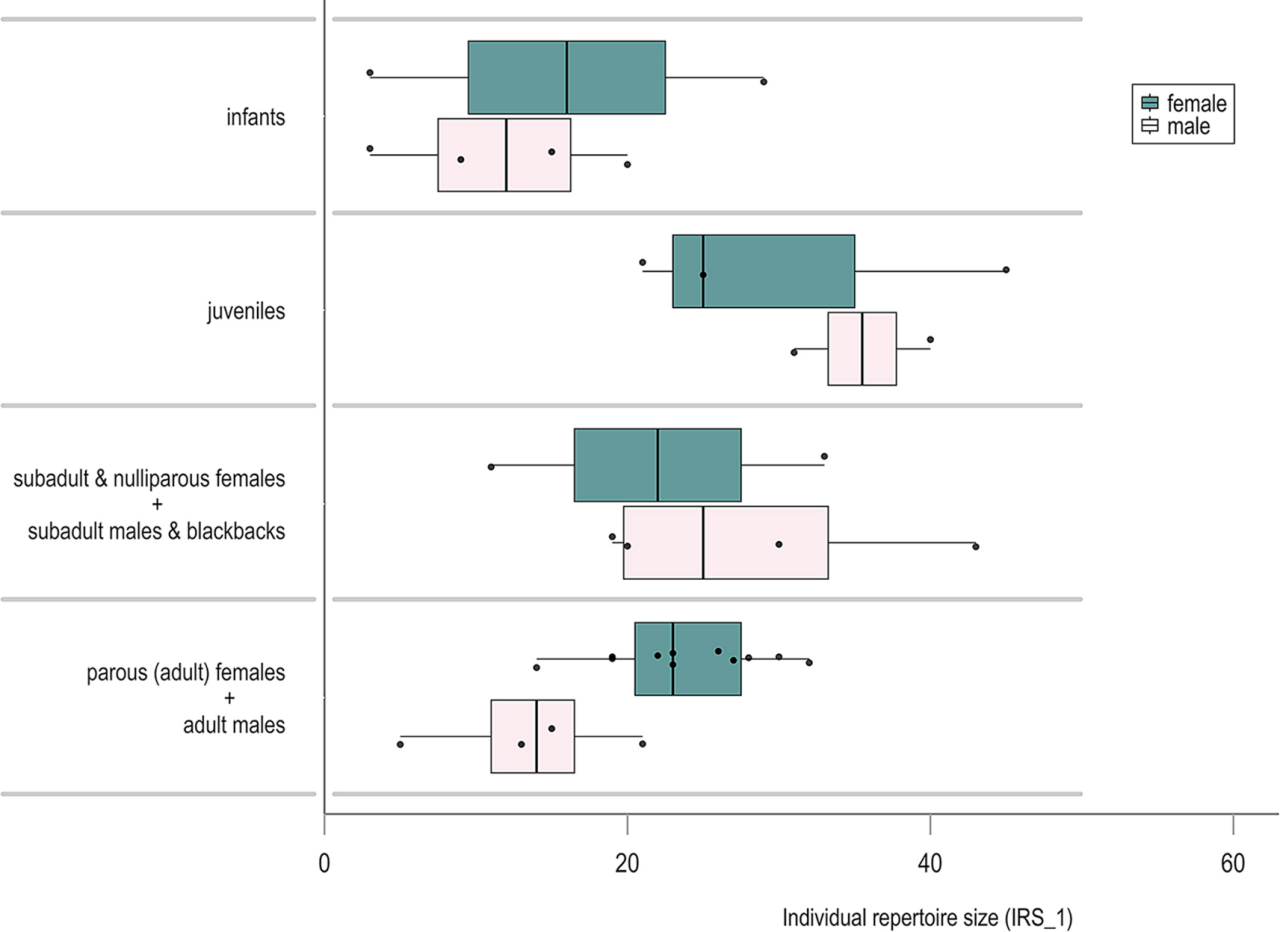
Individual repertoire sizes (IRS_1) across sex and maturation classes. Box plot showing individual repertoire sizes (*n* = 33 data points) across four maturation classes (*cat-1*: infants; *cat-2*: juveniles, *cat-3*: subadults, blackbacks, nulliparous females; *cat-4*: adults) differentiating males and females within each class. IRS values were calculated by considering every gesture action that a given signaller produced as part of their repertoire (even where *n* = 1). Note that 6 out of 27 signallers contributed to more than one maturation class in this comparison

Without applying a threshold for inclusion in an individual gorilla’s repertoire, mean individual repertoire size (*IRS_1*) was 25 gesture actions (sd = 11.2, range = 3–45; *n* = 60 gesture actions; *n* = 2877 gesture tokens; *n* = 27 signallers). Individual gesturing rate (*IGR_obs*) ranged from 0.03 to 1.21 gesture tokens/h of focal group observation time (mean = 0.49, sd = 0.32), and *IRS_1* was positively correlated with the number of gesture tokens observed for each individual (*r*(25) = 0.92, *p* < 0.001; Figure S4). Asymptote was only reached for the silverback MK (at around *n* = 73 gesture tokens, *IRS_1* = 21 gesture actions, *IGR_obs* = 0.51 tokens/h observation time). Setting a threshold for inclusion in the repertoire at *n* = 3 tokens/gesture action/signaller (*IRS_3*), reduced individual repertoires substantially (mean *IRS_3* = 12, sd = 7.1, range = 1–26). The youngest age at which intentional gesture use was observed in the two social units was 8.4 months (BKD, male). The largest average individual repertoire sizes (*IRS_1*) across sex and maturation classes (*n* = 33 data points; 6/27 signallers contributed to more than one class) were present in juveniles (maturation cat-2; mean *IRS_1* = 32.4, sd = 10.0, range = 21–45, *n* = 5 signallers), followed by older not yet fully mature individuals (maturation cat-3: subadults (males and females), blackbacks, nulliparous females; mean *IRS_1* = 27.6, sd = 11.3, range = 11–43, *n* = 7 signallers), then adults (maturation cat-4; mean *IRS_1* = 21.1, sd = 7.2, range = 5–32, *n* = 15 signallers), and infants (maturation cat-1; mean *IRS_1* = 13.2, sd = 10.2, range = 3–29, *n* = 6 signallers). Comparing female and male IRS across maturation classes (see Fig. 4), adult males (*n* = 4 signallers) showed a mean *IRS_1* of 13.5 gesture actions (sd = 6.6, range = 5–21), lower than the average IRS of adult females (mean *IRS_1* = 24, sd = 5.3, range = 14–32, *n* = 11 signallers), and similar to male infants (mean *IRS_1* = 11.8, sd = 7.4, range = 3–20, *n* = 4 signallers). Male juveniles showed the highest IRS in this comparison (mean *IRS_1* = 35.5, range = 31–40, *n* = 2 signallers). More details on IRS values for all maturation and sex classes, including results for *IRS_3* (using a 3 gesture token/gesture action/signaller cut-off to calculate repertoire sizes), are available in Table S14 in the ESM.

Individual gesturing rates (*IGR_obs* and *IGR_vid*) across maturation classes followed an overall similar pattern as repertoire sizes, with the lowest IGR observed in infants (mean *IGR_obs* = 0.36, sd = 0.29, range = 0.04–0.9), followed by adults (mean *IGR_obs* = 0.4, sd = 0.21, range = 0.06–0.7), juveniles (mean *IGR_obs* = 0.85, sd = 0.48, range = 0.38–1.35), and subadults, blackbacks and young nulliparous females (maturation cat-3; mean *IGR_obs* = 0.89, sd = 0.46, range = 0.35–1.6). Adult male IGR (mean *IGR_obs* = 0.24, sd = 0.20, range = 0.06–0.52) was lower than adult female IGR (mean *IGR_obs* = 0.47, sd = 0.19, range = 0.16–0.72) and the highest average IGR value was observed for subadult & nulliparous females (mean *IGR_*obs = 1.2, sd = 0.49, range = 0.68–1.64, females in maturation cat-3, *n* = 3 signallers). For more details on IGR results (including *IGR_vid* (gesture tokens/ h of video material)) as well as IGR results for males and females across maturation classes see Figure S5 and Table S15 in the ESM.

### Contextual diversity in mountain gorilla gesturing

**Fig. 5:**
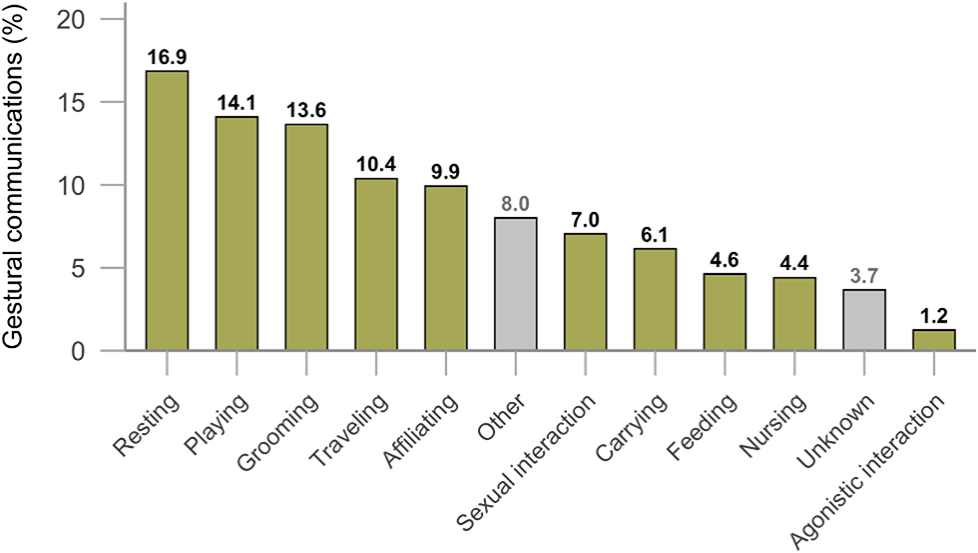
Distribution of Signaller’s context (*n* = 10) following *n* = 1462 gestural communications. Definitions of contexts are listed in Table S8. The group ‘Other’ (in grey) comprises all contexts with a percentage contribution < 1%, the group ‘Unknown’ (in grey) comprises cases where we were unable to estimate the signaller’s behavioural context following a gestural interaction

Mountain gorilla signallers gestured across *n* = 10 broadly defined behavioural contexts (see Fig. 5, *n* = 1462 communications, *n* = 49 signallers).

Communications ended with the signaller resting (16.9%), playing (14.1%), grooming (13.6%), traveling (10.4%), affiliating (9.9%), interacting sexually (7.0%), carrying (6.1%), feeding (4.6%), nursing (4.4%), or being involved in agonistic interaction (1.2%).

### Comparison of ‘new’ gesture actions with descriptions in the wider gorilla literature

Of the 15 newly described mountain gorilla gesture actions (cf. Table 1, Table S16), 10 of them (*Body cross*, *Hit fake*,* Hit non-recipient*,* Hit tab*,* Lay on*,* Lean in*, *Object move: fiddle*,* Object stance*,* Place on object*, and *Turn*) did not resemble previous descriptions in gorilla communication literature (see Table S16 in the ESM for a comparison).

## Discussion

### Gesture form in Bwindi mountain gorillas

Mountain gorillas in the Bwindi Impenetrable National Park employ 63 distinct gesture actions, a similar repertoire size to that described in two studies on wild *Pan* (with comparably large datasets and a similar methodology): Budongo chimpanzees (*n* = 61 gesture types observed 3 or more times; Hobaiter and Byrne 2011a) and Wamba bonobos (*n* = 68 gesture types; Graham et al. 2017). Mountain gorillas showed substantial diversity in gesturing across contexts. Play remained an important context for gesturing; however, as found in other apes when the study of gestural repertoires was extended to wild populations (Hobaiter & Byrne, 2011a; Graham et al. 2017), rather than the overwhelming majority of gestures resulting in play (cf., Genty et al. 2009; where > 70% of gestures were associated with play), wild mountain gorilla gestures regularly resulted in a wide range of social activities, including affiliation, grooming, nursing, carrying, and sexual (incl. socio-sexual) behaviour. We found substantial overlap in gesture form between mountain gorillas’ and other great ape repertoires, with ~ 75% of mountain gorilla core gestural units shared with other ape species. That said, we recorded 15 gesture actions in mountain gorilla communication that may constitute (mountain) gorilla specific forms (with varying degree of certainty): *Body cross*,* Bump into*,* Gaze stance*,* Head avert*,* Hit non-recipient*,* Hit fake*,* Hit tap*,* Jab*,* Lay on*,* Lean in*,* Locomote recipient*,* Object move: fiddle*,* Object stance*,* Place on object*, and *Turn*.

Systematic data across ape species using a similar approach to coding, and in particular on wild (western and eastern) lowland gorilla gestural behaviour, will be extremely valuable in establishing whether these gesture actions do indeed constitute mountain gorilla species-specific gesture forms—or otherwise rare ape-wide, or unrecognised *Gorilla*-wide gesture actions. As a preliminary step—and since similarities with western lowland gorillas are the most likely—we took a close look at comprehensive studies on captive western lowland gorilla gesture (Genty et al. 2009; Pika et al. 2003; Tanner and Byrne 1999), as well as other broader descriptions of gorilla bodily communication in the literature, and tried to establish whether some apparently ‘new’ gesture forms might have been described elsewhere, but masked by differences in approach, such as diverging decisions on splitting and lumping gesture forms (cf. Rodrigues et al. 2021; Grund et al. 2023).

In our comparison of mountain gorilla gestural behaviour with the existing literature, we did not find any descriptions that resemble the relatively frequently used newly-described gesture action *Lean in* (cf. Figure 1c). Predominantly used by adult females towards silverbacks (57%), it appeared to be associated with affiliation. Similarly, *Body cross*,* Hit fake*,* Object move: fiddle*,* Object stance* and *Place on object*, primarily employed in the sexual context, have (to our knowledge) not been described elsewhere. *Lay on*—where the signaller lifts their leg to the side like a dog wanting to urinate and lays it on the recipient’s body (cf. Figure 1b)—while rare, was not matched by any previous descriptions and seems a mountain gorilla specific gesture form. Another rare and particularly interesting case was *Hit non-recipient* (cf. Grund et al. 2024). Here adult females approached (or positioned themselves towards) the silverback, drummed with cupped hands carefully on the back of their (or someone else’s) infant clinging to their chest, while gazing at the silverback (and usually grunting in his direction).

We found possible matches (or partial matches) in descriptions of captive western lowland gorilla gestures for 5/15 of the ‘newly’ described gesture actions: *Bump into*,* Locomote recipient*,* Jab*,* Head avert*, and *Gaze stance*. *Bump into* and *Locomote recipient* could be potentially reflected in the *Pounce* gesture type (Genty et al. 2009), and the mountain gorilla gesture action *Jab* resembled some (but not all) of the example videos for *Punch* (Genty et al. 2009). A similar behaviour to *Head avert* (mountain gorillas) is mentioned in the description of the *Stiff walk* gesture type (definition: ‘walking with rigid forelegs and usually head tilted on the side’; Genty et al. 2009); however, here the head tilt was not coded separately and the equivalent *Locomote stiff* in the mountain gorillas was frequently not accompanied by the gesture action *Head avert* (although *Head avert* was typically accompanied by a stiff body posture). While the mountain gorilla gesture action *Gaze stance*—characterised by the signaller positioning their body and face towards the recipient, holding it in a stiff posture and gazing intently at the recipient’s face/eyes—could potentially be a combination of the gesture types *Look* (definition: ‘staring intensely at another individual’) together with *Stiff stance* (definition: ‘standing rigidly with stiff limbs and forelimbs held tight, facial expression of tight lips usually occurs in sexual context’) described by Genty et al. (2009), the behaviour depicted in the available example videos does not resemble the mountain gorilla *Gaze stance*, and the *Look* is closer to descriptions of peering (cf. Schuppli et al. 2016). That said, *Gaze stance* closely matches descriptions of body postures and communicative gaze reported in studies on sexual solicitation behaviour in captive western lowland gorillas (Atsalis et al. 2004; Stoinski et al. 2009) and Virunga mountain gorillas (Watts 1991; Yamagiwa 1992; Nadler 1989), where it is often referred to as ‘staring’ (e.g., Watts 1991; Yamagiwa 1992; Atsalis et al. 2004). These cases highlight the substantial difficulty in comparing across studies using different methods where gesture units were defined in full a priori, and in interpreting movement from written descriptions. While we do not advocate for the use of a single approach to coding—the method used should reflect the questions of interest (and often the data available; Rodrigues et al. 2021)—there are advantages to using fine-grained component-based approaches to coding, from which different units can be (re)constructed based on current, and future, interests (Grund et al. 2023).

Interestingly, the gesture action *Present*—not reported in descriptions of (captive) western lowland gorilla gesturing (Genty et al. 2009; Pika et al. 2003; Tanner and Byrne (1999), but a very common gesture in *Pan* gesturing (e.g., Hobaiter and Byrne 2011a; Graham et al. 2017)—was the second most frequently observed gesture action in mountain gorillas. While in mountain gorillas *Presents* were primarily employed to initiate grooming, they were also observed in mother-offspring dyads, where mothers communicated to their infants to climb on or to nurse (similarly to chimpanzees and bonobos: Graham et al. 2018). As adult western lowland gorillas generally rarely—if ever—groom each other (Masi et al. 2009; Robbins et al. 2016), the absence of *Presents* to initiate grooming is perhaps unsurprising; however, mother-offspring interactions should typically include similar communicative behaviour in western lowland and mountain gorillas. Thus, the absence of the gesture action *Present* seems unlikely to be fully explained by differences in communicative affordances between the species.

In contrast, the gesture action *Clap*, regularly reported across studies of captive western lowland gorillas (Genty et al. 2009; Pika et al. 2003; Tanner and Byrne 1999), and one of the few gesture actions regularly described in wild western lowland gorillas (Fay 1989; Salmi and Muñoz 2020; Kalan and Rainey 2009), appears absent in Bwindi mountain gorillas (see also Robbins et al. 2016). In the wild, *Clap* is described as being used by females to alert the silverback to potential danger (Salmi and Muñoz 2020), indirectly promoting group cohesion (Kalan and Rainey 2009), as well as to initiate play (Salmi and Muñoz 2020). As mountain gorillas are more socially cohesive than their western lowland counterparts, they may have less need of a salient acoustic signal to alert group members.

Communicative adjustments to the diverse and flexible socio-ecological conditions experienced by ape species may be expressed not only at the level of the gesture action, but more subtly, for example, in finer-grained morphological units such as the gesture morphs. Of 45 gesture actions with sufficient cases for analysis, 26 showed at least two variants, resulting in 63 additional gestural units in the repertoire (totalling 126 units). Interestingly, the degree of splitting observed in the mountain gorilla morph repertoire is very similar to that in a recent study on Eastern chimpanzee gesturing employing similar modifiers (Mielke et al. 2024). Here 42/61 gesture actions had sufficient cases for analysis, 25 showed at least one variant (*n* = 73 additional units), totalling 134 units in the Eastern chimpanzee morph repertoire (115 units from the morph analysis with an additional 19 unspecified gesture actions (< 10 observations). With morphs as the unit in the repertoire, we may find more subtle variation in repertoire expression not only across, but also potentially within the same species— for example, depending on interactant pairs and/or associated social goals. The Eastern chimpanzee morph units in Mielke et al. (2024) were slightly more accurate in predicting the outcome of a gestural interaction (i.e., more goal-specific) as compared to their respective gesture actions. In the future, we could similarly reassess the mountain gorilla morph units by considering their goal or meaning as the core property by which signals are lumped or split (cf., Hobaiter and Byrne 2017; Mielke et al. 2024). However, describing a signal repertoire from the perspective of the number of goals or meanings may limit our understanding of social nuances in its use. For example, in human languages, variation in form can indicate a shift in meaning, but can also reflect variation in accent, dialect, or prosody. Defining units based solely on semantic shift, may limit our ability to detect fine-grained cultural variations in expression of the same meaning. In other apes, as in humans, unit meaning may change situationally based on the social and/or situational context (Graham et al. 2020; Hobaiter et al. 2022; Bohn et al. 2022; Franke et al. 2024) and can be influenced by other signals produced in combination (Genty et al. 2014; Genty 2019; Hobaiter et al. 2017; Wilke et al. 2017 et al. 2019). To make things even more complicated, our ability to detect and categorise apes’ communicative goals remains in its infancy (e.g., Hobaiter et al. 2022), while concurrently, the splitting of units based on semanticity would be heavily influenced by exactly how broad vs. fine-grained we were able (or decided) to differentiate between specific goals.

Ultimately, the level of repertoire splitting may best be chosen depending on the question being asked and the availability of additional information that could be of importance when analysing/interpreting the interaction as a whole. The GesturalOrigins mountain gorilla gesture coding that underlies this study offers future possibilities to flexibly factor in different structural modifiers as well as other variables such as the situational context, the social relationship of the interactants, co-occurring vocal and facial signals, and the outcome of the communicative interaction. While we are confident that we were able to catalogue mountain gorilla gestural movements (gesture actions, e.g., *Raise*) and additional structural features (modifiers – e.g., body part = *Arm*) in a systematic way—offering the first comprehensive description of mountain gorilla gestural behaviour—we do not see the presented repertoire as fixed or exhaustive. As long-lived animals with a highly flexible social system, additional (perhaps rare) gestural movements may be observed in Bwindi and elsewhere.

### Bwindi mountain gorilla individual repertoire sizes and gesturing rate

Mountain gorillas’ average individual repertoire size of 12 gesture actions (mean *IRS_3*) lies between that reported for wild chimpanzees (*n* = 10, Hobaiter and Byrne 2011a) and bonobos (*n* = 14.4, Graham et al. 2017). The average repertoire size of adult mountain gorillas (mean *IRS_3 =* 9.9 gesture actions, ~ 16% of the overall repertoire) was twice that reported for wild adult chimpanzees (5.1 gesture actions, ~ 8% of the overall repertoire, Hobaiter and Byrne 2011a), whereas juvenile repertoires were similarly large (mean *IRS_3* = 14.6; juvenile chimpanzees: *n* = 15.1, Hobaiter and Byrne 2011a). Unlike in chimpanzees (infant chimpanzee mean repertoire size = 7.9, Hobaiter and Byrne 2011a), the smallest repertoires (with a mean *IRS_3* of 4.3 gesture actions) were observed in infants, and not in adults. Thus, while the broad trend towards juvenile apes using a larger variety of gesture actions as compared to adults or infants holds (e.g., *siamangs*: Liebal et al. 2004a; *orangutans*: Liebal et al. 2006; *chimpanzees*: Liebal et al. 2004b; Hobaiter and Byrne 2011a; *gorillas*: Genty et al. 2009), it seemed less pronounced in mountain gorillas.

As in previous wild ape gesture studies, mountain gorilla individual repertoire sizes varied greatly (ranging from 1 to 26 when considering all ages, and 1 to 18 in adults, *IRS_3*), and were strongly correlated with the number of gesture instances reported for each individual, with individual ape’s repertoires in mountain gorillas, as in other apes (Hobaiter and Byrne 2011a; Graham 2016), rarely reaching asymptote. While this suggests that sampling efforts are an important limiting factor on our ability to describe individual repertoires, the weak association between repertoire size and observation time combined with the variability in individual gesturing rates, suggests sampling density may have varying impact on individual repertoire size, and could be influenced by age (see also Fröhlich et al. 2017). For example, gesturing rate was highest in maturation class 3 (subadult males and females + blackbacks + nulliparous females; *IRS_obs* = 0.89), but individuals in this category did not show the largest individual repertoire sizes. Similarly, Mugwere (MG), Korugyzei (KR), Bwebisha (BS), and Mukiza (MK), all adult individuals (three females and one silverback) of the same social unit (MUK), showed very similar repertoire sizes (between 21 and 23 gesture actions), but substantial variation in their appearance in the recorded video material (with MG for example only appearing in 17 h and MK, the most frequently occurring individual, in 52 h of video material). The repertoire size of the least recorded individual (Kadogo (KD); *n* = 5 h) and that of the most frequently recorded individual MK varied by only 5 gesture actions (KD-*IRS_1* = 16, MK-*IRS_1* = 21).

But—even where complete—what does repertoire size really tell us? Some researchers suggest that a larger signal repertoire size reflects a need for communicative nuance to navigate more complex and diverse social environments (Freeberg et al. 2012). Mountain gorillas’ relatively large adult repertoires (adult males mean *IRS_1* = 13.5; adult females mean *IRS_1* = 24) could reflect the substantial variation and flexibility in the organisation of their social units. Adult females may have to be particularly flexible in their communicative expression while navigating different social environments—they can experience multiple secondary transfer events within their lifetime (Robbins and Robbins 2015), in the course of which they associate with different silverbacks and reside in groups of varying social composition (e.g., in multi-male and one-male groups). Interestingly, in the vocal domain adult males (silverbacks) show larger signal repertoires than adult females (Salmi et al. 2013). Some acoustic signals are able to transmit over great distances, even in visually dense environments (e.g., Mitani and Stuht 1998). This extended range may be particularly helpful for silverbacks, given their need for both within-group and inter-group signalling (Wright et al. 2021; Robbins and Sawyer 2007; Salmi and Doran-Sheehy 2014; Perlman and Salmi 2017). While some gestural signals also seem to serve a broadcast function (e.g., the acoustically salient adult male chest-beat, Wright et al. 2021), the majority of gestural signalling occurs within close-range dyadic interaction, with gesture use tailored to the specific recipient, context, and goal. Mountain gorilla females face numerous nuanced communicative challenges: including competition with other females over the attention of the resident silverback (Watts 1992, 1994; Doran-Sheehy et al. 2009; Morrison et al. 2023), and (given sexual dimorphism) the need to persuade a much larger social partner to achieve their goals. As a result they may be particularly skilled gestural negotiators. Supporting this line of thought, individual gesturing rates were twice as high in adult females (mean *IGR_obs* = 0.47 gestures/h) than in adult males (mean *IGR_obs* = 0.24 gestures/h), a trend that was also observable in the comparison of subadult males + blackbacks (mean *IGR_obs* = 0.67 gestures/h) vs. subadult + (young) nulliparous females (mean *IGR_obs* = 1.19 gestures/h). Interestingly, a particularly high *IGR_obs* value in the subadult and nulliparous female category (the individual BS) was driven mainly by requests for sexual interaction, suggesting female sexual solicitation behaviour may be a particularly prolific context of mountain gorilla gesturing (as for male chimpanzees during consortship, Hobaiter and Byrne 2012). While anecdotal, identifying social goals associated with high gesturing rates and/or repertoire sizes, highlights social needs that are prominently solved via gestures, and may underly some of the observed deviations in individual gesturing rates and repertoires sizes across maturation classes. On the other hand, repertoire size and gesturing rate may also reflect the development of social competences. The large gestural repertoires consistently reported in great ape juveniles and subadults (e.g., *orangutans*: Liebal et al. 2006; *chimpanzees*: Liebal et al. 2004b; Hobaiter and Byrne 2011a; *gorillas*: Genty et al. 2009, present study), have been suggested to be the result of younger individuals exploring and fine-tuning their use of the very large available repertoire (Hobaiter and Byrne 2011b).

With ~ 6 gesture tokens/h observation time, Bwindi mountain gorillas seem to be more prolific gesturers than wild western lowland gorillas, particularly considering the relatively scarce gestural data researchers were able to obtain for wild western lowland gorillas, despite considerable data collection effort (cf. Genty et al. 2009).

However, the estimated average gesturing rate per individual of 0.49 gestures/h observation time (including infants) is much lower than estimations of individual gesturing rates of wild Eastern chimpanzees (Budongo Forest: Hobaiter et al. 2017; mean 3.9 gestural signals/h of focal). Thus, while gestural interaction plays a prominent role in wild mountain gorilla daily life and possibly more so than in western lowland gorillas, individual gesturing rates seem to be higher in *Pan*. Future research analysing the structure of gestural communication events, such as sequence lengths and/or response latencies may be able to disentangle whether this is due to chimpanzees simply having more social requests than mountain gorillas, being more communicative when requesting social goals, or, perhaps, chimpanzee recipients being less responsive/signallers more persistent (since signallers faced with less responsive recipients, may tend to employ more gestures within the same communication to achieve their goal, given they persist in the goal). In a first preliminary comparison of Bwindi mountain gorilla and Budongo chimpanzee response latencies for the outcome *Grooming* requested with the gesture action *Present*, mountain gorilla recipients responded more slowly than chimpanzees to grooming requests (Grund et al. 2023). However, how persistent and/or responsive signallers and recipients tend to be, will also likely depend on the valence and relative importance of a particular social goal, which likely differs across species, social relationships, and contexts. *Grooming*, for example, has particularly important social valence in chimpanzees’ hierarchical fission-fusion societies, where social bonds (mediated via grooming interaction) affect a male’s position in the social hierarchy, with potential knock on effects on fitness, including mating access (Muller and Mitani 2005; Wroblewski et al. 2009; Feldblum et al. 2021), and competition around who grooms (and is groomed by) whom, can be expected (Watts 2000). Communicative requests for the goal *Grooming*, while also frequent in mountain gorilla gesturing (cf. Figure 5), may thus look structurally different in chimpanzees as compared to mountain gorillas. Other less everyday communicative contexts, for example requests for sexual interaction, which interestingly were associated with a range of potentially species-specific gesture actions in mountain gorillas, may also show different structural patterns in other aspects of communication (e.g., persistence and response latencies).

All extant ape species show both similarities in body plan and the way they use their bodies and minds to communicate intentionally, and substantial differences in social structure, sociality, and in socio-ecological niche. Much of the inquiry into great ape cognitive abilities, and in particular their linguistic capacities, has centred heavily on *Pan* (in particular chimpanzees; Rodrigues et al. 2021). While no modern ape species is a perfect model for ancestral states in the human or ape lineage (Lameira and Call 2020), mountain gorillas, living in comparatively small cohesive social units, characterised by relatively stable female-male bonding, and with variable and fluid patterns of social association, make an essential contribution to a well-rounded understanding of the social features that shape ape communication. With this study we begin to bridge long-standing gaps in wild ape gesture research, providing crucial additional context for our theories on how ape communication—including human language—evolved.

## Electronic supplementary material

Below is the link to the electronic supplementary material.

Supplementary Material 1

## Data Availability

Partial anonymised data and the code used for analyses are available on GitHub (https://github.com/CharlotteGrund/mg_repertoire). Full datasets are available from the corresponding author upon reasonable request. Gesture action example videos are accessible on Zenodo (10.5281/zenodo.13341597).

## References

[CR1] Arbib M (2012) How the brain got language: the mirror neuron hypothesis. Oxford University Press, Oxford, UK

[CR2] Atsalis S, Margulis SW, Bellem A, Wielebnowski N (2004) Sexual behavior and hormonal estrus cycles in captive aged lowland gorillas (*Gorilla gorilla*). Am J Primatol 62(2):123–132. 10.1002/ajp.20010 10.1002/ajp.20010 14983470

[CR3] Badihi G, Graham KE, Grund C, Safryghin A, Soldati A, Donnellan E, Hobaiter C (2024) Chimpanzee gestural exchanges share Temporal structure with human Language. Curr Biol 34(14):R673–R674. 10.1016/j.cub.2024.06.009 10.1016/j.cub.2024.06.009 39043136

[CR4] Bard KA (1992) Intentional behavior and intentional communication in young free-ranging orangutans. Child Dev 63(5):1186–1197. 10.1111/j.1467-8624.1992.tb01688.x 1446548

[CR5] Bohn M, Liebal K, Oña L, Tessler MH (2022) Great ape communication as contextual social inference: a computational modelling perspective. Philosophical Trans Royal Soc B 377(1859):20210096. 10.1098/rstb.2021.0096 10.1098/rstb.2021.0096 PMC9310183 35876204

[CR6] Breuer T, Hockemba MBN, Olejniczak C, Parnell RJ, Stokes EJ (2009) Physical maturation, life-history classes and age estimates of free‐ranging Western gorillas—Insights from Mbeli bai, Republic of congo. Am J Primatology: Official J Am Soc Primatologists 71(2):106–119. 10.1002/ajp.20628 10.1002/ajp.20628 19003901

[CR7] Byrne RW, Cartmill E, Genty E, Graham KE, Hobaiter C, Tanner J (2017) Great ape gestures: intentional communication with a rich set of innate signals. Anim Cogn 20(4):755–769. 10.1007/s10071-017-1096-4 10.1007/s10071-017-1096-4 PMC5486474 28502063

[CR8] Canteloup C, Bovet D, Meunier H (2015) Intentional gestural communication and discrimination of human attentional States in rhesus macaques (*Macaca mulatta*). Anim Cogn 18:875–883. 10.1007/s10071-015-0856-2 10.1007/s10071-015-0856-2 25749401

[CR9] Cartmill EA, Byrne RW (2007) Orangutans modify their gestural signaling according to their audience’s comprehension. Curr Biol 17(15):1345–1348. 10.1016/j.cub.2007.06.069 10.1016/j.cub.2007.06.069 17683939

[CR09] Cartmill EA, Byrne RW (2010) Semantics of primate gestures: intentional meanings of orangutan gestures. Anim Cognit 13:793–804. 10.1007/s10071-010-0328-7 10.1007/s10071-010-0328-7 20563619

[CR10] Cheney DL, Seyfarth RM (2018) Flexible usage and social function in primate vocalizations. Proceedings of the National Academy of Sciences 115(9):1974–1979 10.1073/pnas.1717572115 PMC5834704 29432157

[CR11] Corballis MC (2009) Language as gesture. Hum Mov Sci 28(5):556–565. 10.1016/j.humov.2009.07.003 10.1016/j.humov.2009.07.003 19665811

[CR12] Crockford, Boesch C (2005) Call combinations in wild chimpanzees. Behaviour 142(4):397–421. http://www.jstor.org/stable/4536251

[CR13] Doran-Sheehy DM, Fernández D, Borries C (2009) The strategic use of sex in wild female Western gorillas. Am J Primatology: Official J Am Soc Primatologists 71(12):1011–1020. 10.1002/ajp.20743 10.1002/ajp.20743 19722225

[CR14] ELAN (Version 6.4) [Computer software]. (2022). Nijmegen: Max Planck Institute for Psycholinguistics, The Language Archive. Retrieved from https://archive.mpi.nl/tla/elan

[CR15] Fay JM (1989) Hand-clapping in Western lowland gorillas (*Gorilla gorilla gorilla*). Mammalia 53(3):457–458. 10.1515/mamm.1989.53.3.457

[CR17] Feldblum JT, Krupenye C, Bray J, Pusey AE, Gilby IC (2021) Social bonds provide multiple pathways to reproductive success in wild male chimpanzees. Iscience 24(8). 10.1016/j.isci.2021.102864 10.1016/j.isci.2021.102864 PMC8390850 34471859

[CR16] Franke M, Bohn M, Fröhlich M (2024) Latent meaning representations in great-ape gestural communication. In Proceedings of the Annual Meeting of the Cognitive Science Society (Vol. 46). https://escholarship.org/uc/item/56c0m5t0

[CR18] Freeberg TM, Dunbar RI, Ord TJ (2012) Social complexity as a proximate and ultimate factor in communicative complexity. Philosophical Trans Royal Soc B: Biol Sci 367(1597):1785–1801. 10.1098/rstb.2011.0213 10.1098/rstb.2011.0213 PMC3367695 22641818

[CR20] Fröhlich M, Wittig RM, Pika S (2016) Play-solicitation gestures in chimpanzees in the wild: flexible adjustment to social circumstances and individual matrices. Royal Soc Open Sci 3(8):160278. 10.1098/rsos.160278 10.1098/rsos.160278 PMC5108953 27853603

[CR19] Fröhlich M, Müller G, Zeiträg C, Wittig RM, Pika S (2017) Gestural development of chimpanzees in the wild: the impact of interactional experience. Anim Behav 134:271–282. 10.1016/j.anbehav.2016.12.018

[CR21] Fröhlich M, Bartolotta N, Fryns C, Wagner C, Momon L, Jaffrezic M, van Schaik CP (2021) Orangutans have larger gestural repertoires in captivity than in the wild—A case of weak innovation? Iscience 24(11). 10.1016/j.isci.2021.103304 10.1016/j.isci.2021.103304 PMC8601978 34820602

[CR24] Genty E (2019) Vocal–gestural combinations in infant bonobos: new insights into signal functional specificity. Anim Cogn 22(4):505–518. 10.1007/s10071-019-01267-0 10.1007/s10071-019-01267-0 31098849

[CR22] Genty E, Breuer T, Hobaiter C, Byrne RW (2009) Gestural communication of the gorilla (*Gorilla gorilla*): repertoire, intentionality and possible origins. Anim Cogn 12(3):527–546. 10.1007/s10071-009-0213-4 10.1007/s10071-009-0213-4 PMC2757608 19184669

[CR23] Genty E, Clay Z, Hobaiter C, Zuberbühler K (2014) Multi-modal use of a socially directed call in bonobos. PLoS ONE 9(1):e84738. 10.1371/journal.pone.0084738 10.1371/journal.pone.0084738 PMC3893130 24454745

[CR25] Goodall J (1986) The chimpanzees of gombe: patterns of behavior. Cambridge University Press

[CR26] Graham KE (2016) Meaning and context in the gestural communication of wild bilia (bonobo: Pan paniscus). Doctoral dissertation, University of St Andrews

[CR30] Graham KE, Hobaiter C (2023) Towards a great ape dictionary: inexperienced humans understand common nonhuman ape gestures. PLoS Biol 21(1):e3001939. 10.1371/journal.pbio.3001939 10.1371/journal.pbio.3001939 PMC9873169 36693024

[CR28] Graham KE, Furuichi T, Byrne RW (2017) The gestural repertoire of the wild bonobo (*Pan paniscus*): a mutually understood communication system. Anim Cogn 20(2):171–177. 10.1007/s10071-016-1035-9 10.1007/s10071-016-1035-9 PMC5306194 27632158

[CR31] Graham KE, Hobaiter C, Ounsley J, Furuichi T, Byrne RW (2018) Bonobo and chimpanzee gestures overlap extensively in meaning. PLoS Biol 16(2):e2004825. 10.1371/journal.pbio.2004825 10.1371/journal.pbio.2004825 PMC5828348 29485994

[CR29] Graham KE, Furuichi T, Byrne RW (2020) Context, not sequence order, affects the meaning of bonobo (*Pan paniscus*) gestures. Gesture 19(2–3):335–364. 10.1075/gest.19028.gra

[CR27] Graham KE, Badihi G, Safryghin A, Grund C, Hobaiter C (2022) A socio-ecological perspective on the gestural communication of great ape species, individuals, and social units. Ethol Ecol Evol 34(3):235–259. 10.1080/03949370.2021.1988722 10.1080/03949370.2021.1988722 PMC9067943 35529671

[CR32] Gruber T, Clay Z (2016) A comparison between bonobos and chimpanzees: A review and update. Evolutionary Anthropology: Issues News Reviews 25(5):239–252. 10.1002/evan.21501 10.1002/evan.21501 27753219

[CR33] Grueter CC, Robbins AM, Abavandimwe D, Vecellio V, Ndagijimana F, Ortmann S, Stoinski TS, Robbins MM (2016) Causes, mechanisms, and consequences of contest competition among female mountain gorillas in Rwanda. Behav Ecol 27(3):766–776. 10.1093/beheco/arv212

[CR34] Grund C, Badihi G, Graham KE, Safryghin A, Hobaiter C (2023) GesturalOrigins: A bottom-up framework for Establishing systematic gesture data across ape species. Behav Res Methods 56(2):986–1001. 10.3758/s13428-023-02082-9 10.3758/s13428-023-02082-9 PMC10830607 36922450

[CR35] Grund C, Robbins MM, Hobaiter C (2024) Mountain gorilla gesture action example videos [Data set]. Zenodo. 10.5281/zenodo.13341597

[CR36] Gupta S, Sinha A (2019) Gestural communication of wild Bonnet macaques in the Bandipur National park, Southern India. Behav Process 168:103956. 10.1016/j.beproc.2019.103956 10.1016/j.beproc.2019.103956 31493494

[CR37] Halina M, Rossano F, Tomasello M (2013) The ontogenetic ritualization of bonobo gestures. Anim Cogn 16:653–666. 10.1007/s10071-013-0601-7 10.1007/s10071-013-0601-7 23370783

[CR39] Harcourt AH, Stewart KJ (2007) Gorilla society: what we know and don’t know. Evolutionary Anthropology: Issues News Reviews 16(4):147–158. 10.1002/evan.20142

[CR38] Harcourt AH, Hauser M, Stewart KJ (1993) Functions of wild Gorilla ‘close’ calls. I. Repertoire, context, and interspecific comparison. Behaviour 124(1–2):89–122. 10.1163/156853993X00524

[CR40] Hedwig D, Hammerschmidt K, Mundry R, Robbins MM, Boesch C (2014) Acoustic structure and variation in mountain and Western gorilla close calls: A syntactic approach. Behaviour 151(8):1091–1120. 10.1163/1568539X-00003175

[CR41] Hewes GW (1973) Primate communication and the gestural origins of Language. Curr Anthropol 14:5–24. http://www.jstor.org/stable/2741093

[CR43] Hobaiter C, Byrne RW (2011a) The gestural repertoire of the wild chimpanzee. Anim Cogn 14(5):745–767. 10.1007/s10071-011-0409-2 21533821

[CR44] Hobaiter C, Byrne RW (2011b) Serial gesturing by wild chimpanzees: its nature and function for communication. Anim Cogn 14:827–838. 10.1007/s10071-011-0416-3 10.1007/s10071-011-0416-3 21562816

[CR45] Hobaiter C, Byrne RW (2012) Gesture use in consortship: wild chimpanzees’ use of gesture for an ‘evolutionarily urgent’purpose. In Developments in primate gesture research (pp. 129–146). John Benjamins Publishing Company. 10.1075/gs.6.08hob

[CR46] Hobaiter C, Byrne RW (2014) The meanings of chimpanzee gestures. Curr Biol 24(14):1596–1600. 10.1016/j.cub.2014.05.066 10.1016/j.cub.2014.05.066 24998524

[CR47] Hobaiter C, Byrne RW (2017) What is a gesture? A meaning-based approach to defining gestural repertoires. Neurosci Biobehavioral Reviews 82:3–12. 10.1016/j.neubiorev.2017.03.008 10.1016/j.neubiorev.2017.03.008 29229064

[CR48] Hobaiter C, Byrne RW, Zuberbühler K (2017) Wild chimpanzees’ use of single and combined vocal and gestural signals. Behav Ecol Sociobiol 71:1–13. 10.1007/s00265-017-2325-1 10.1007/s00265-017-2325-1 PMC5446553 28596637

[CR42] Hobaiter C, Badihi G, Daly, Gabriela BDM, Eleuteri V, Graham KE, Grund C, Henderson M, Rodrigues ED, Safryghin A, Soldati A, Wiltshire C (2021) The Great Ape Dictionary video database (1.0.0) [Data set]. Zenodo. 10.5281/zenodo.5600472

[CR49] Hobaiter C, Graham KE, Byrne RW (2022) Are ape gestures like words? Outstanding issues in detecting similarities and differences between human Language and ape gesture. Philosophical Trans Royal Soc B 377(1860):20210301. 10.1098/rstb.2021.0301 10.1098/rstb.2021.0301 PMC9358316 35934962

[CR50] Kalan AK, Rainey HJ (2009) Hand-clapping as a communicative gesture by wild female swamp gorillas. Primates 50:273–275. 10.1007/s10329-009-0130-9 10.1007/s10329-009-0130-9 19221858

[CR51] Kano T (1982) The social group of pygmy chimpanzees (*Pan paniscus*) of Wamba. Primates 23(2):171–188. 10.1007/BF02381159

[CR52] Kano T (1992) The last ape: pygmy chimpanzee behavior and ecology, vol 155. Stanford University Press, Stanford. 10.1007/BF02220263

[CR53] Kersken V, Gómez JC, Liszkowski U, Soldati A, Hobaiter C (2019) A gestural repertoire of 1-to 2-year-old human children: in search of the ape gestures. Anim Cogn 22:577–595. 10.1007/s10071-018-1213-z 10.1007/s10071-018-1213-z PMC6647402 30196330

[CR54] Knox A, Markx J, How E, Azis A, Hobaiter C, van Veen FJF, Morrogh-Bernard H (2019) Gesture use in communication between mothers and offspring in wild Orang-Utans (*Pongo Pygmaeus wurmbii*) from the Sabangau Peat-Swamp forest, Borneo. Int J Primatol 40(3):393–416. 10.1007/s10764-019-00095-w

[CR55] Lameira AR, Call J (2020) Understanding Language evolution: beyond pan-centrism. BioEssays 42(3):1900102. 10.1002/bies.201900102 10.1002/bies.201900102 31994246

[CR56] Leavens DA, Russell JL, Hopkins WD (2010) Multimodal communication by captive chimpanzees (*Pan troglodytes*). Anim Cogn 13(1):33–40. 10.1007/s10071-009-0242-z 10.1007/s10071-009-0242-z PMC2797826 19504272

[CR57] Leroux M, Chandia B, Bosshard AB, Zuberbühler K, Townsend SW (2022) Call combinations in chimpanzees: a social tool? Behav Ecol 33(5):1036–1043. 10.1093/beheco/arac074

[CR58] Liebal K, Pika S, Tomasello M (2004a) Social communication in Siamangs (*Symphalangus syndactylus*): use of gestures and facial expressions. Primates 45(1):41–57. 10.1007/s10329-003-0063-7 10.1007/s10329-003-0063-7 14655035

[CR59] Liebal K, Call J, Tomasello M (2004b) Use of gesture sequences in chimpanzees. Am J Primatology: Official J Am Soc Primatologists 64(4):377–396. 10.1002/ajp.20087 10.1002/ajp.20087 15580580

[CR60] Liebal K, Pika S, Tomasello M (2006) Gestural communication of orangutans (*Pongo pygmaeus*). Gesture 6(1):1–38. 10.1075/gest.6.1.02lie

[CR61] Luef EM, Liebal K (2013) The hand-on gesture in gorillas (*Gorilla gorilla*). Interact Stud 14(1):44–61. 10.1075/is.14.1.04lue

[CR62] Manguette ML, Robbins AM, Breuer T, Stokes EJ, Parnell RJ, Robbins MM (2020) Female dispersal patterns influenced by male tenure duration and group size in Western lowland gorillas. Behav Ecol Sociobiol 74:1–15. 10.1007/s00265-020-02863-8

[CR63] Masi S, Cipolletta C, Robbins MM (2009) Western lowland gorillas (*Gorilla gorilla gorilla*) change their activity patterns in response to frugivory. Am J Primatology: Official J Am Soc Primatologists 71(2):91–100. 10.1002/ajp.20629 10.1002/ajp.20629 19021124

[CR64] McNeilage A, Plumptre AJ, Brock-Doyle A, Vedder A (2001) Bwindi impenetrable National park, uganda: Gorilla census 1997. Oryx 35(1):39–47. 10.1046/j.1365-3008.2001.00154.x

[CR65] Mielke A, Badihi G, Graham KE, Grund C, Hashimoto C, Piel AK, Hobaiter C (2024) Many morphs: parsing gesture signals from the noise. Behav Res Methods 1–18. 10.3758/s13428-024-02368-6 10.3758/s13428-024-02368-6 PMC11362259 38438657

[CR66] Mitani JC, Stuht J (1998) The evolution of nonhuman primate loud calls: acoustic adaptation for long-distance transmission. Primates 39:171–182. 10.1007/BF02557729

[CR67] Molesti S, Meguerditchian A, Bourjade M (2020) Gestural communication in Olive baboons (*Papio anubis*): repertoire and intentionality. Anim Cogn 23(1):19–40. 10.1007/s10071-019-01312-y 10.1007/s10071-019-01312-y 31605248

[CR68] Morrison RE, Dunn JC, Illera G, Walsh PD, Bermejo M (2020) Western gorilla space use suggests territoriality. Sci Rep 10(1):3692. 10.1038/s41598-020-60504-6 10.1038/s41598-020-60504-6 PMC7067784 32165643

[CR69] Morrison R, Ndayishimiye E, Stoinski T, Eckardt W (2023) Female age and reproductive stage influence copulation patterns in mountain gorillas’ variable mating system. Behav Ecol Sociobiol 77(6):72. 10.1007/s00265-023-03346-2

[CR70] Mountain Gorilla Census—New numbers announced! - Gorilla Doctors. (2019), December 16 Gorilla Doctors. https://www.gorilladoctors.org/new-mountain-gorilla-numbers/

[CR71] Muller MN, Mitani JC (2005) Conflict and Cooperation in wild chimpanzees. Adv Study Behav 35:275–331. 10.1016/S0065-3454(05)35007-8

[CR72] Nadler RD (1989) Sexual initiation in wild mountain gorillas. Int J Primatol 10:81–92. 10.1007/BF02736246

[CR73] Nishida T (1968) The social group of wild chimpanzees in the Mahali mountains. Primates 9:167–224. 10.1007/BF01730971

[CR74] Oña LS, Sandler W, Liebal K (2019) A stepping stone to compositionality in chimpanzee communication. PeerJ 7:e7623. 10.7717/peerj.7623 10.7717/peerj.7623 PMC6745191 31565566

[CR75] Ostrofsky KR, Robbins MM (2020) Fruit-feeding and activity patterns of mountain gorillas (*Gorilla Beringei Beringei*) in Bwindi impenetrable National park, Uganda. Am J Phys Anthropol 173(1):3–20. 10.1002/ajpa.24056 10.1002/ajpa.24056 32274796

[CR92] Perlman M, Salmi R (2017) Gorillas May use their laryngeal air sacs for whinny-type vocalizations and male display. J Lang Evol 2(2):126–140. 10.1093/jole/lzx012

[CR76] Pika S, Liebal K, Tomasello M (2003) Gestural communication in young gorillas (*Gorilla gorilla*): gestural repertoire, learning, and use. Am J Primatology: Official J Am Soc Primatologists 60(3):95–111. 10.1002/ajp.10097 10.1002/ajp.10097 12874841

[CR77] Pika S, Liebal K, Tomasello M (2005) Gestural communication in subadult bonobos (*Pan paniscus*): repertoire and use. Am J Primatology: Official J Am Soc Primatologists 65(1):39–61. 10.1002/ajp.20096 10.1002/ajp.20096 15645456

[CR78] Pollick AS, De Waal FB (2007) Ape gestures and Language evolution. Proc Natl Acad Sci 104(19):8184–8189. 10.1073/pnas.0702624104 10.1073/pnas.0702624104 PMC1876592 17470779

[CR79] Robbins AM, Robbins MM (2015) Dispersal patterns of females in the genus *Gorilla*. In: Furuichi T, Yamagiwa J, Aureli F (eds) Dispersing primate females. Primatology monographs. Springer, Tokyo. 10.1007/978-4-431-55480-6_4

[CR82] Robbins MM, Robbins AM (2018) Variation in the social organization of gorillas: life history and socioecological perspectives. Evolutionary Anthropology: Issues News Reviews 27(5):218–233. 10.1002/evan.21721 10.1002/evan.21721 30325554

[CR81] Robbins MM, Sawyer S (2007) Intergroup encounters in mountain gorillas of Bwindi impenetrable National park, Uganda. Behaviour 144(12):1497–1519. http://www.jstor.org/stable/4536530

[CR80] Robbins AM, Stoinski T, Fawcett K, Robbins MM (2009) Leave or conceive: Natal dispersal and philopatry of female mountain gorillas in the virunga volcano region. Anim Behav 77(4):831–838. 10.1016/j.anbehav.2008.12.005

[CR83] Robbins MM, Ando C, Fawcett KA, Grueter CC, Hedwig D, Iwata Y, Lodwick JL, Masi S, Salmi R, Stoinski TS, Todd A, Vercellio V, Yamagiwa J (2016) Behavioral variation in gorillas: evidence of potential cultural traits. PLoS ONE 11(9):e0160483. 10.1371/journal.pone.0160483 10.1371/journal.pone.0160483 PMC5014408 27603668

[CR84] Robbins MM, Ortmann S, Seiler N (2022) Dietary variability of Western gorillas (*Gorilla gorilla gorilla*). PLoS ONE 17(8):e0271576. 10.1371/journal.pone.0271576 10.1371/journal.pone.0271576 PMC9401121 36001558

[CR85] Robbins MM, Akantorana M, Arinaitwe J, Breuer T, Manguette M, McFarlin S, Robbins AM (2023) Comparative life history patterns of female gorillas. Am J Biol Anthropol 181(4):564–574. 10.1002/ajpa.24792 10.1002/ajpa.24792 37345324

[CR87] Roberts AI, Vick SJ, Roberts SGB, Buchanan-Smith HM, Zuberbühler K (2012) A structure-based repertoire of manual gestures in wild chimpanzees: statistical analyses of a graded communication system. Evol Hum Behav 33(5):578–589. 10.1016/j.evolhumbehav.2012.05.006

[CR86] Roberts AI, Roberts SGB, Vick SJ (2014) The repertoire and intentionality of gestural communication in wild chimpanzees. Anim Cogn 17:317–336. 10.1007/s10071-013-0664-5 10.1007/s10071-013-0664-5 23999801

[CR88] Rodrigues ED, Santos AJ, Veppo F, Pereira J, Hobaiter C (2021) Connecting primate gesture to the evolutionary roots of language: A systematic review. Am J Primatol 83(9):e23313. 10.1002/ajp.23313 10.1002/ajp.23313 34358359

[CR89] Salmi R, Doran-Sheehy DM (2014) The function of loud calls (Hoot Series) in wild Western gorillas (*Gorilla gorilla*). Am J Phys Anthropol 155(3):379–391. 10.1002/ajpa.22575 10.1002/ajpa.22575 25059429

[CR90] Salmi R, Muñoz M (2020) The context of chest beating and hand clapping in wild Western gorillas (*Gorilla gorilla gorilla*). Primates 61(2):225–235. 10.1007/s10329-019-00782-5 10.1007/s10329-019-00782-5 31894436

[CR91] Salmi R, Hammerschmidt K, Doran-Sheehy DM (2013) Western Gorilla vocal repertoire and contextual use of vocalizations. Ethology 119(10):831–847. 10.1111/eth.12122

[CR93] Schel AM, Bono A, Aychet J, Pika S, Lemasson A (2022) Intentional gestural communication amongst red-capped mangabeys (*Cercocebus torquatus*). *Animal Cognition*, 1–18. 10.1007/s10071-022-01615-7 10.1007/s10071-022-01615-7 PMC9617956 35362785

[CR94] Schuppli C, Meulman EJ, Forss SI, Aprilinayati F, van Noordwijk MA, van Schaik CP (2016) Observational social learning and socially induced practice of routine skills in immature wild orang-utans. Anim Behav 119:87–98. 10.1016/j.anbehav.2016.06.014

[CR95] Slocombe KE, Zuberbühler K (2005) Functionally referential communication in a chimpanzee. Curr Biol 15(19):1779–1784. 10.1016/j.cub.2005.08.068 10.1016/j.cub.2005.08.068 16213827

[CR97] Stoinski TS, Perdue BM, Legg AM (2009) Sexual behavior in female Western lowland gorillas (*Gorilla gorilla gorilla*): evidence for sexual competition. Am J Primatology: Official J Am Soc Primatologists 71(7):587–593. 10.1002/ajp.20692 10.1002/ajp.20692 19399838

[CR96] Stokes EJ, Parnell RJ, Olejniczak C (2003) Female dispersal and reproductive success in wild Western lowland gorillas (*Gorilla gorilla gorilla*). Behav Ecol Sociobiol 54:329–339. 10.1007/s00265-003-0630-3

[CR98] Tanner JE, Byrne RW (1999) The development of spontaneous gestural communication in a group of zoo-living lowland gorillas. In The Mentalities of Gorillas and Orangutans: Comparative Perspectives (pp. 211–239). Cambridge University Press. 10.1017/CBO9780511542305.012

[CR99] Tomasello M, George BL, Kruger AC, Jeffrey M, Farrar, Evans A (1985) The development of gestural communication in young chimpanzees. J Hum Evol 14(2):175–186. 10.1016/S0047-2484(85)80005-1

[CR100] Villa-Larenas F, Llorente M, Liebal K, Amici F (2024) Gestural communication in wild spider monkeys (*Ateles geoffroyi*). Anim Cogn 27(1):18. 10.1007/s10071-024-01854-w 10.1007/s10071-024-01854-w PMC10907450 38429467

[CR101] Watts DP (1985) Relations between group size and composition and feeding competition in mountain gorilla groups. Anim Behav 33(1):72–85. 10.1016/S0003-3472(85)80121-4

[CR102] Watts DP (1991) Mountain gorilla reproduction and sexual behavior. Am J Primatol 24(3–4):211–225. 10.1002/ajp.1350240307 10.1002/ajp.1350240307 31952383

[CR103] Watts DP (1992) Social relationships of immigrant and resident female mountain gorillas. I. Male-female relationships. Am J Primatol 28(3):159–181. 10.1002/ajp.1350280302 10.1002/ajp.1350280302 31941211

[CR104] Watts DP (1994) Agonistic relationships between female mountain gorillas (*Gorilla gorilla beringei*). Behav Ecol Sociobiol 34(5):347–358. 10.1007/BF00197005

[CR105] Watts DP (2000) Grooming between male chimpanzees at ngogo, Kibale National park. II. Influence of male rank and possible competition for partners. Int J Primatol 21(2):211–238. 10.1023/A:1005421419749

[CR106] Wilke C, Kavanagh E, Donnellan E, Waller BM, Machanda ZP, Slocombe KE (2017) Production of and responses to unimodal and multimodal signals in wild chimpanzees, *Pan troglodytes schweinfurthii*. Anim Behav 123:305–316. 10.1016/j.anbehav.2016.10.024

[CR107] Wright E, Grawunder S, Ndayishimiye E, Galbany J, McFarlin SC, Stoinski TS, Robbins MM (2021) Chest beats as an honest signal of body size in male mountain gorillas (*Gorilla Beringei Beringei*). Sci Rep 11(1):6879. 10.1038/s41598-021-86261-8 10.1038/s41598-021-86261-8 PMC8032651 33833252

[CR108] Wroblewski EE, Murray CM, Keele BF, Schumacher-Stankey JC, Hahn BH, Pusey AE (2009) Male dominance rank and reproductive success in chimpanzees, *Pan troglodytes schweinfurthii*. Anim Behav 77(4):873–885. 10.1016/j.anbehav.2008.12.014 10.1016/j.anbehav.2008.12.014 PMC2689943 19498952

[CR109] Yamagiwa J (1992) Functional analysis of social staring behavior in an all-male group of mountain gorillas. Primates 33(4):523–544. 10.1007/BF02381153

[CR110] Yamagiwa J, Kahekwa J, Basabose AK (2003) Intra-specific variation in social organization of gorillas: implications for their social evolution. Primates 44(4):359–369. 10.1007/s10329-003-0049-5 10.1007/s10329-003-0049-5 12942370

[CR111] Young C, Robbins MM (2023) Association patterns of female gorillas. Philosophical Trans Royal Soc B 378(1868):20210429. 10.1098/rstb.2021.0429 10.1098/rstb.2021.0429 PMC9703218 36440560

