# Supplementary material for: The gestural repertoire of Bwindi mountain gorillas (Gorilla beringei beringei): gesture form and frequency of use
Source: Anim Cogn. 2025 Jul 29;28(1):73. doi: 10.1007/s10071-025-01977-8 (PMC12307521; doi:10.1007/s10071-025-01977-8)
Supplement: Supplementary file 1 — Supplementary Material 1 [file 10071_2025_1977_MOESM1_ESM.docx]

**Electronic supplementary material (ESM)**

The manuscript’s electronic supplementary material (along with code and partial datasets) is also available on GitHub: <https://github.com/CharlotteGrund/mg_repertoire>).

*Article title:* **The gestural repertoire of Bwindi mountain gorillas (*Gorilla beringei beringei*): gesture form and frequency of use**

*Authors:* Charlotte Grund^1^, Martha M. Robbins^2^, Catherine Hobaiter^1^

^1^ School of Psychology and Neuroscience, University of St Andrews, St Andrews, UK

^2^ Department of Primate Behavior and Evolution, Max Planck Institute for

Evolutionary Anthropology, Leipzig, Germany

*Corresponding author:* Charlotte Grund (grund.charlotte[at]gmx.de)

*Journal*: Animal Cognition

**Table S1. Bitukura: individuals, sex, age, and date of birth.**

| **No** | **Individual** | **Code** | **Sex** | **Day** | **Month** | **Year** | **Est.** |
| --- | --- | --- | --- | --- | --- | --- | --- |
| 1 | Batahi | BTH | m | *28* | *7* | 2021 | n |
| 2 | Betina^*^ | BT | f | *-* | *-* | 1992 | y |
| 3 | Kadogo | KD | m | *-* | *-* | 2005 | y |
| 4 | Kakuto | KT | m | 7 | 4 | 2013 | n |
| 5 | Kamuga | KG | f | *-* | *-* | 1992 | y |
| 6 | Kanoel | NL | f | 28 | 12 | 2011 | n |
| 7 | Kirabo | KB | f | - | - | 2016 | n |
| 8 | **Mugisha** | **GH** | m | *-* | *-* | 1998 | y |
| 9 | Nyiguru | NG | m | 11 | 10 | 2015 | n |
| 10 | Ruhara | RH | f | *-* | *-* | 1988 | y |
| 11 | ***Rukumu*** | ***RK*** | m | *-* | *-* | 1988 | Y |
| 12 | Rungo | RG | m | 4 | 2 | 2011 | n |
| 13 | Tangara | TAN | m | 13 | 5 | 2021 | n |
| 14 | Thursday | TD | f | 22 | 9 | 2005 | n |
| * transferred to MUK on 14/07/2021. Est. = Estimated birth year; n = no, y = yes; silverbacks are in bold font (note that Rukumu (RK) is subordinate to GH and of old age). | | | | | | | |

**Table S2. Mukiza: individuals, sex, age, and date of birth.**

| **No** | **Individual** | **Code** | **Sex** | **Day** | **Month** | **Year** | **Est.** |
| --- | --- | --- | --- | --- | --- | --- | --- |
| 1 | Bakundine | BKD | m | 17 | 2 | 2020 | n |
| 2 | Betina^*^ | BT | f | *-* | *-* | 1992 | y |
| 3 | Bwebisha | BS | f | *-* | *-* | 2009 | y |
| 4 | Gorodi | GO | f | 4 | 2 | 2016 | n |
| 5 | Kagote | GTE | m | *23* | *1* | 2018 | n |
| 6 | Kanywani^#^ | KY | m | 16 | 6 | 2011 | n |
| 7 | Korugyzei | KR | f | *-* | *-* | 1996 | y |
| 8 | Kwetegyeka | KTG | m | 10 | 10 | 2020 | n |
| 9 | Mubwindi | BW | f | 19 | 2 | 2008 | n |
| 10 | Mugenyi | JN | f | *-* | *-* | 2001 | y |
| 11 | Mugwere | MG | f | *-* | *-* | 1989 | y |
| 12 | **Mukiza** | **MK** | m | 29 | 11 | 1999 | n |
| 13 | Nyakabara | NY | f | 1 | 6 | 2017 | n |
| 14 | Rutooma | RTM | m | *31* | *1* | 2019 | n |
| 15 | Tonvi | TV | f | 5 | 7 | 2013 | n |
| 16 | Kajungu | KJU | m | 23 | 1 | 2022 | n |
| 17 | Twijukye | TW | f | *-* | *-* | 2000 | y |
| * transferred from BIT to MUK in 07/2021; ^#^ disappeared in 2021; Est. = Estimated birth year; n = no, y = yes. | | | | | | | |

**Table S3. Oruzogo: individuals, sex, age, and date of birth.**

| **No** | **Individual** | **Code** | **Sex** | **Day** | **Month** | **Year** | **Est.** |
| --- | --- | --- | --- | --- | --- | --- | --- |
| 1 | ***Bwengye*** | ***BGY*** | m | *-* | *-* | 2003 | y |
| 2 | **Kaganga** | **KGG** | m | *-* | *-* | 2000 | y |
| 3 | **Kasumali** | **KSM** | m | *-* | *-* | 2001 | y |
| 4 | Katoto | KTO | f | *-* | *-* | 2008 | y |
| 5 | Mufuriki | MFK | m | 11 | 11 | 2014 | n |
| 6 | Mukwano | MKW | f | *-* | *-* | 2005 | y |
| 7 | Munaba | MB | f | 6 | 11 | 2011 | n |
| 8 | Musana | SAN | f | 17 | 1 | 2017 | n |
| 9 | Musenene | MNE | m | *-* | *-* | 2011 | y |
| 10 | Mutesi | MTS | f | *-* | *-* | 1995 | y |
| 11 | Ngabirano | GBR | m | *-* | *-* | 2011 | y |
| 12 | Nyakashunju | NKJ | m | *-* | *-* | 2011 | y |
| 13 | Nyakina | NKN | f | *-* | *-* | 1995 | y |
| 14 | Nyangaro | NYG | f | *-* | *-* | 1990 | y |
| 15 | Paska | PSK | m | 18 | 4 | 2017 | n |
| 16 | Rukeija | RKJ | m | *-* | *-* | 2007 | y |
| 17 | Sabaato | SBT | m | 25 | 7 | 2020 | n |
| Est. = Estimated birth year; n = no, y = yes; silverbacks are in bold font. Note that Oruzogo was only followed sporadically mainly due to different regulations during the Covid-19 pandemic. The social unit split during the study period. The data from FS 3 is from 12 individuals that associated with KSL and BGY. BGY took over the group when KSL died in 2022 (shortly after data collection ended). | | | | | | | |

**Table S4. Rukara: individuals, sex, age, and date of birth.**

| **No** | **Individual** | **Code** | **Sex** | **Day** | **Month** | **Year** | **Est.** |
| --- | --- | --- | --- | --- | --- | --- | --- |
| 1 | Bajurizi | AJ | m | 3 | 6 | 2017 | n |
| 2 | Happy | HP | m | 1 | 9 | 2005 | n |
| 3 | Kengongwe | GG | m | 31 | 12 | 2010 | n |
| 4 | Korubaro | BR | m | 15 | 9 | 2011 | n |
| 5 | Munaba | MB | f | 6 | 11 | 2011 | n |
| 6 | **Rukara** | **RR** | m | *-* | *-* | 1996 | y |
| 7 | Tindamanyire | TN | f | *-* | *-* | 1994 | y |
| Est. = Estimated birth year; n = no, y = yes; silverback is in bold font. During the last field season (FS 3) Rukara group consisted of: AJ, GG, BR, RR and occasionally HP. TN and MB had transferred to other social units (MB on 14/08/2020 to Oruzogo; TN on 13/02/2021 to Kyagurilo B (social unit in habituation)). | | | | | | | |

**Table S5. Data collection periods.**

| Data collection | Dates | Months | Observation days |
| --- | --- | --- | --- |
| Pilot | August 2019 | 1 | 17 |
| Season 1 | 01/12/2019 – 17/03/2020 | 3.5 | 59 |
| Season 2 | 01/07/2021 – 31/08/2021 | 2 | 45 |
| Season 3 | 20/01/2022 – 24/03/2022 | 2 | 33 |

**Table S6. Observation time and amount of video data collected in each group.**

Daily research time was limited to maximally 4 h in each group following UWA regulations. Daily observation time of gorillas in the different groups varied further depending on tourist visits and at what time in the day gorillas were found (there is no possibility to collect data after 2 pm, so if gorillas were e.g., found at 10 am and tourists were still visiting the group the maximum amount of research time available was 3 h). During the Covid-19 pandemic the social units RUK and ORU were visited less often. Observation effort for BIT and MUK remained balanced.

| **Group** | **Days** | **T_Obs (h)** | **Vid (h)** | **Gesture tokens** | **n Sgn** |
| --- | --- | --- | --- | --- | --- |
| **Mukiza** | 63 | 225.8 | 94.4 | 1602 | 16 |
| **Bitukura** | 60 | 220.0 | 80.8 | 1370 | 12 |
| **Rukara** | 16 | 60.4 | 20.8 | 167 | 7 |
| **Oruzogo** | 15 | 46.4 | 23.0 | 139 | 16 |
| **TOTAL** | **154*** | **552.7** | **218.9** | **3278** | **49^+^** |
| T Obs (h) = observation time in hours; Vid (h) = recorded video footage in hours; Gesture tokens = number of instances of intentional gesture use; n Sgn = number of signallers.* the total number of observation days here adds up to 154, however, gorillas were visited on 151 days (on 3 days more than one social unit was subject to data collection due to natural encounters). ^+^ the total number of unique signallers is 49 individuals (and not 51) since two individuals (BT and MB) transferred between groups. | | | | | |

**Gesture coding**

*Intentionality* and *social directedness* were evaluated by looking at the *gaze*, *contact,* and *response waiting* behaviour of the signaller (e.g., Liebal et al., 2004a). We pre-classified signals as being potentially socially directed (at a specific recipient) if the signaller clearly gazed at the recipient before (and/or during) signalling. For signals that established physical contact with another individual (e.g., *Hit recipient* or *Grab*), gaze before or during were not required to pre-classify the signal as being directed towards a specific individual. In both cases subsequent *response waiting* after signalling (i.e., monitoring the recipient and waiting for a response for at least 1 second) was then required for the interaction to be considered intentional gesturing. In one rare gesture action (*Hit non-recipient*), the individual that was gazed at and the individual that was contacted were not aligned (the signaller made contact with one individual while gazing (and directing other behaviour) towards another). For this (rare) gesture action, we considered gaze to overrule contact, and these gestures were considered to be directed to the individual the signaller gazed at during signalling (for definition of the gesture action *Hit non-recipient* see Table 1 in results section). This follows a similar method to discriminate gestures extended towards one individual while gazing at another during possible examples of pointing (Hobaiter et al., 2014).

**Figure S1. Coding gestural behaviour with the GesturalOrigins template.** Example gesture action coded in ELAN (screenshot). The upper left panel shows the video that is analysed. Below to the left there is a panel with all the tiers that can be selected and coded (the coding template). Note that only a fraction of the available tiers is captured by the screenshot. The tiers are organised hierarchically and are either free text or use single-select predetermined ‘controlled vocabulary’. We explain the coding procedure in detail in this video tutorial (see also Grund et al., 2023): <https://www.youtube.com/watch?v=VoShxy1vYe4&list=PLAidTwBvwFPkRdZ2OMVntnfmfOgozomr6&index=43>

| 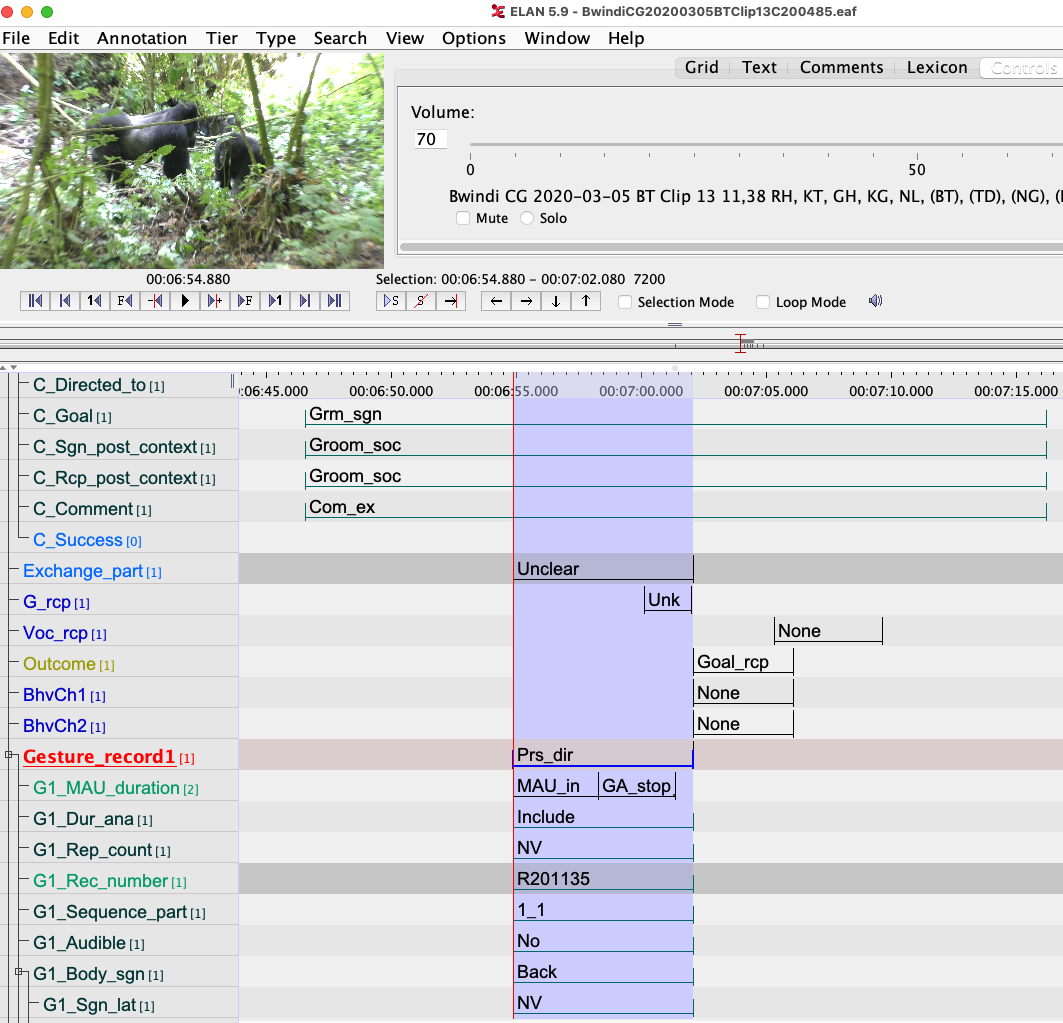 |
| --- |

Repertoire construction: gesture actions, modifiers, and morphs

Our bottom-up approach to coding gesture aims to minimise *a priori* structural choices to systematically examine gestural units in the repertoire at different levels of resolution post-coding – from fine-grained and highly-split, to lumped and more inclusive categories. Figure S2 below illustrates the bottom-up coding and the subsequent morph analysis.

**Figure S2. Scheme illustrating the bottom-up coding and the subsequent morph detection.** For each instance of gesture use we first coded the basic gestural movement observed (*Gesture action* (GA)). The physical form of that gesture action instance was further specified by the coding of modifier variables (usually multiple options, e.g., different body parts in the Body part modifier). After finishing the coding process, data were compiled and analysed for consistent configurations of modifier levels within each gesture action (Morph analysis). A gesture action might have several morphs, i.e., variants (illustrated here by the splitting of the light green rectangle and light red circle with a solid line – the gesture actions - into smaller subsets), or may only ever occur in one particular configuration (here the triangle).


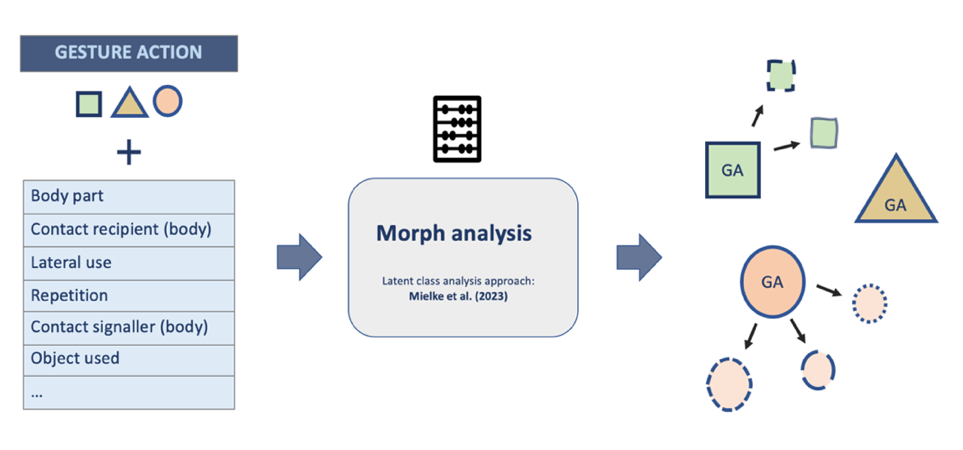


We tailored the methodological approach used by Mielke et al. (2024) for Eastern chimpanzees to include modifiers appropriate for mountain gorilla gesture use, allowing us to explore additional (potentially meaningful) levels of data-driven repertoire units within the mountain gorilla gestural system. Typically, only very rare levels of modifiers were lumped to supra-categories from the original coding. Doing so balances the relevancy of the modifier in the gestural system (in terms of for how many gesture instances it was coded for) with the amount of detail it provides. Table S7 below lists the modifiers included in the present analysis, together with the level at which they were coded and analysed, and their relevance for different subsets of gesture actions.

**Table S7. Gesture action modifiers and their levels considered in the morph detection (n = 45 gesture actions).**

| **Modifier** | **Description** | **Gesture actions** | **Original level options** | **Levels in coded data (original)** | **Levels in coded data (lumped)** |
| --- | --- | --- | --- | --- | --- |
| Body part | The body part the signaller used to perform the gestural movement (valid for all gesture actions). | (all ) | Arm, Back, BackShoulder, Body, BodyChest, BodyFront, Bottom, BottomGenitalskin, Face, Fingers, Fist, Foot, Hand, HandFoot, HandWrist, HeadHead, HeadThroatNeck, Knuckles, Leg, Mouth, MouthTeeth, Penis, Swelling, Testes | Arm, Back, BackShoulder, Body, BodyChest, BodyFront, Bottom, BottomGenitalskin, Face, Fingers, Fist, Foot, Hand, HandFoot, HandWrist, HeadHead, Knuckles, Leg, Mouth, MouthTeeth, Penis | Arm, Back, Body, BodyChest, BodyFront, Bottom, Fingers, Fist, Foot, Genitals, Hand, HandFoot, Head, Knuckles, Leg, Mouth |
|  | Total n levels | N = 45 | N = 24 | N = 21 | N = 16 |
| Contact recipient | The area of the recipient’s body the signaller contacted as part of the gestural action (valid for contact gesture actions) | Bite, Bump into, Embrace, Grab, Grab hold, Hit recipient, Hit recipient soft, Hit tap, Jab, Kiss, Lean in, Pull, Push, Stroke, Touch, Touch long | *same as above* | Arm, Back, BackShoulder, Body, BodyChest, BodyFront, Bottom, BottomGenitalskin, Face, Fingers, Foot, Hand, HandWrist, HeadHead, HeadThroatNeck, Knuckles, Leg, Mouth, Penis | Extremity, Genitals, Head, Mouth, TorsoBack, TorsoFront |
|  |  | N = 16 | N = 24 | N = 19 | N = 6 |
| Lateral use | Valid for gesture actions that involve limb use (e.g., Grab) and where there is variability in the number of extremities involved (e.g. 1 vs. 2 Hand(s), 1 vs. 2 Arm(s) etc.) – how was the gesture instance performed, unimanual or bimanual (lateral use of extremities) ? | Chest beat, Chest beat informal, Embrace, Fling, Grab, Grab hold, Hit object, Hit recipient, Hit recipient soft, Hit self, Hit tap, Object move, Object shake, Pull, Push, Shake, Stomp object, Stroke, Swing | Bimanual, Unimanual | Bimanual, Unimanual | Bimanual, Unimanual |
|  |  | N = 19 | N = 2 | N = 2 | N = 2 |
| Repetition | Valid for gesture actions with elements that are at times rhythmically repeated (e.g., HitObject or HitSelf). Did the gesture instance involve rhythmical repetition or was it singly employed? | Hit object, Hit recipient, Hit recipient soft, Hit self, Hit tap, Object shake, Shake, Spin pirouette, Stomp object, Stroke, Swing | *free text* | 1-30 | Singular, Repeated |
|  |  | N = 11 | - | - | N = 2 |

In principle, a modifier can only have an effect in the LCA morph analysis if there was non-negligible variation in its levels for a specific gesture action (i.e., if the coded gesture tokens of the respective gesture action showed more than one of the potential levels of the modifier in question, and at least 5 occurrences of each).

Note that in the GesturalOrigins approach we code several additional structural features (‘modifiers’) that could potentially be relevant shapers of great ape gestures (e.g., the object type used to perform the gesture action, the flexion status of the limbs in free-limb gesture actions, or even the speed of gesture action performance) and that could be included in the morph analysis (e.g., in an alternative level of resolution or in a more fine-grained one); however we decided to limit the number of modifiers for two main reasons.

Firstly, we were working with a new species, which means there was very little previous information on species-specific salient features of the gestural behaviour that we could incorporate in (pre-morph analysis) decisions. Secondly, while we coded over 3000 instances of signal use, data density within gesture actions varied and adding more modifiers (or modifier levels) would have made computational analysis for most of them unstable or impossible. We therefore prioritised those modifiers relevant across the widest range of gesture actions (note that, with the exception of the modifier *Body* part, coded for all gesture actions, most modifiers are specific to a subset of gesture actions. For example, the *Contact recipient* modifier (which part of the recipient’s body was contacted) is of course only relevant for gesture actions that establish contact with the recipient.).

Signaller context

The variable “Context post (signaller)” describes the behavioural context of the signaller after the gestural interaction (entire communication). Table S8 offers a definition for each of the behavioural contexts. For the current analysis we lumped some of the originally coded context levels (e.g., *Feeding (monopolisable)* was lumped with *Feeding*, *Resting (contact)* was lumped *Resting*, and *Socio-sexual activity* with *Sexual activity*) *–* for more details on the original levels and the variable itself see Grund et al (2023)).

**Table S8. Context definitions (levels).**

| **Context** | **Description** |
| --- | --- |
| Affiliating | The individual is engaged in a form of affiliative behaviour (prosocial contact that is none of the behaviours specified below) |
| Agonistic | The individual is engaged in a negative social interaction, (includes mild forms, such as cough grunting or displays (as the signaller or the recipient), but also contact aggression) |
| Carrying | The individual is either carried by or carrying another individual |
| Feeding | The individual is feeding (note that if the individual was engaged in a social activity at the same time (e.g., being groomed by another individual, or in the case of mothers, nursing offspring, the social activity took precedence) |
| Grooming | The individual is either grooming or being groomed by another individual |
| Nursing | The individual is either nursing from or nursed by another individual (mother offspring interaction) |
| Playing | The individual is engaged in play interaction. |
| Resting | The individual is resting (with or without being in contact with another individual). |
| Sexual interaction | Includes *Socio-sexual activity* (The individual is mounting or being mounted by another individual and there is pelvic thrusting/movement and the behaviour occurs between individuals that are not potentially reproductive (immature individuals or same-sex adults) and *Sexual activity* (The individual is copulating or just copulated (mature males and subadult/adult females, i.e., potentially reproductive sexual activity)) |
| Traveling | The individual is in locomotion (includes climbing trees). Mountain gorillas typically travel rather slowly. Note that whenever the individual started to travel after a feeding bout and was still feeding while travelling (but not stopping again) this was coded as travelling. |

Note that there were two additional levels ‘Playing (solitary)’ and ‘Grooming (solitary)’ that were observed, but they constituted < 1 % of the observed contexts. Whenever the context was unclear (visible but rare or miscellaneous, i.e., none of the above), it was assigned the level ‘Other’. Whenever the signaller was not visible following the gestural communication the context was ‘Unknown’.

**Interrater reliability**

We tested 5% of the data (only including gesture actions with at least 10 observations), i.e., n = 170 gesture tokens. To ensure that we tested a representative sample, we ran a function that randomly selected record numbers (i.e., tokens) to a sample size that was proportional to each gesture action’s representation in the data. The respective annotations in the corresponding ELAN files were erased by C.G. and the variables recoded by C.H. Statistical tests (Cohen’s kappa) on the data were conducted by a third person (K.E. Graham).

Note that *Directed to, Gaze*, and *Response waiting* variables were skewed as the result of focusing our coding on intentional gestures. As Kappa tests are substantially impacted by data skew, we provide percentage agreement in addition to aid in their interpretation. Agreement in the relevant variables was substantial to perfect throughout (see Table S9 below). For full descriptions of each variable see the GesturalOrigins coding protocol (Grund et al., 2023).

Coding agreement of very rarely recorded gesture actions (< 10 observations) was not directly tested; however, we verified gesture action classifications and definitions by looking at at least three video examples of each gesture action and resolving any differences through discussion.

**Table S9. Interobserver reliability test of included variables**

| **Variable** | **Cohen’s kappa (% agreement)** | **Test data** |
| --- | --- | --- |
| Directed to | κ = 0.64 (83.4%) | raw |
| Gesture action (record) | κ = 0.88 | raw |
| Repetition count | κ = 1.0 | lumped |
| Body part signaller | κ = 0.89 | raw |
| Signaller laterality | κ = 0.93 | raw |
| Laterality bimanual | κ = 0.78 | raw |
| Body part contacted | κ = 0.80 | raw |
| Contact laterality | κ = 1.0 | raw |
| Gaze before | κ = 0.64 (95.8%) | raw |
| Gaze during | κ = 0.83 (96.1%) | raw |
| Response waiting | κ = 0.91 (99.2%) | raw |
| Context post (signaller) | κ = 0.87 | raw |

**Table S10. MAU definitions of potentially mountain gorilla specific gesture actions (cf. video examples: Grund et al. 2024).** We describe and categorise the physical form of gestural behaviour after the basic bodily movement (‘gesture action’) that the signaller performs. Here each gesture action is defined (prior to coding) on the basis of a ‘minimal action unit’ (MAU, Grund et al. 2023), the part of the gestural movement that sufficiently discriminates it from any other action type in the gestural system.

**
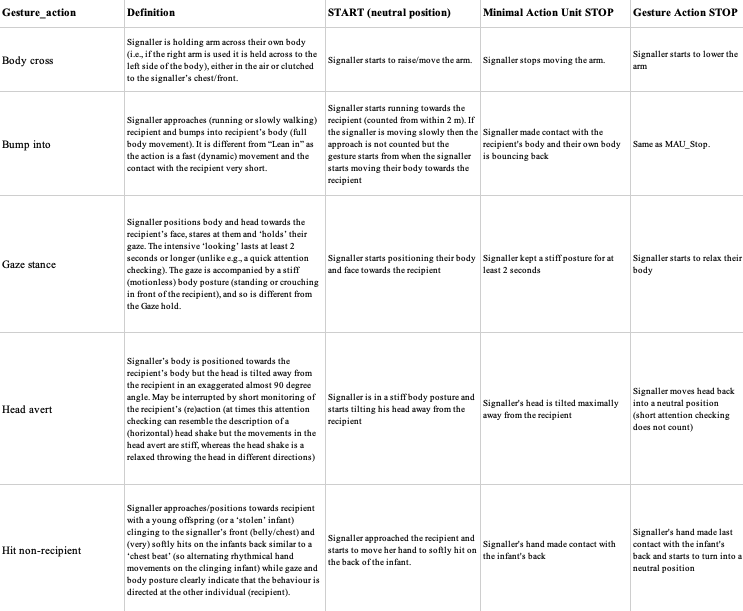
**

**
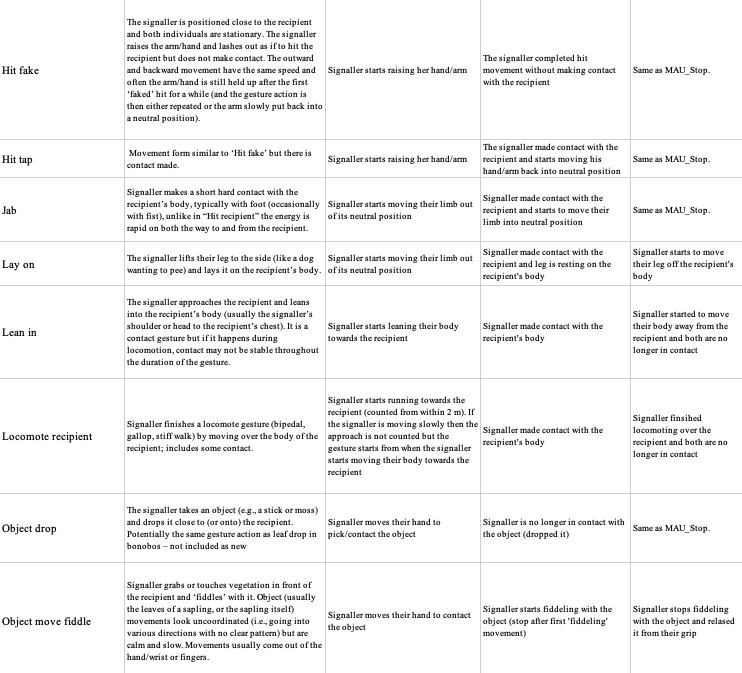
**

**_
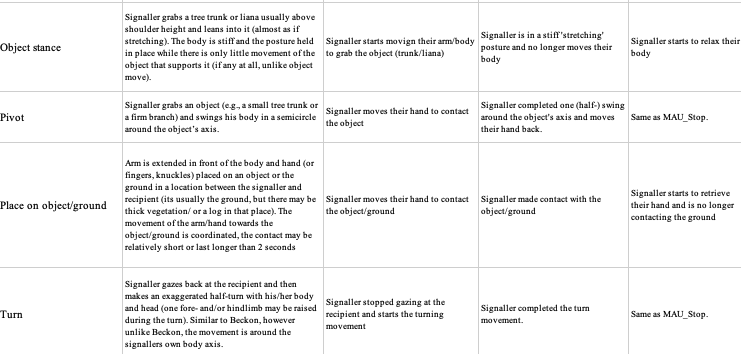
_**

**Table S11. Definitions of great ape gesture actions and newly identified units.** The repertoire of great ape gesture actions is taken from Grund et al. (2023), based on systematic descriptions in the cross-species comparison Byrne et al. (2017), and combined with the new gesture actions described in this study (newly identified gesture actions are indicated with asterisks). The column P indicates the presence or absence of gesture actions in the mountain gorilla repertoire. Gesture actions considered as part of the mountain gorilla repertoire (observed on at least three occasions) are indicated with **1**. Gesture actions that were observed on fewer than three occasions are indicated with **(1)**. Gesture actions seen in at least one other ape species but not seen in mountain gorillas are indicated with **0**. Italic font in the gesture definition column indicates additional information not necessarily part of the gesture action definition (comments relating to categorisation). More detailed definitions of newly identified gesture actions, including MAU start and stops are presented in Table S10. For MAU definitions of all other gesture actions listed see Grund et al. (2023).

| **Gesture action** | **P** | **Gesture definition** |
| --- | --- | --- |
| Beckon | **1** | A scooping movement from one or more of the joints (e.g., fingers, wrist, elbow), movement extends towards recipient and then back to the signaller in one active motion. |
| Big loud scratch | **(1)** | Loud exaggerated scratching movement on signaller's own body (must not be followed by self-grooming). A single BLS might include a small change in movement angle; but if contact stops or body part changes code as new BLS. *There is one possible occasion of the use of a big loud scratch gesture action in the mountain gorillas, but the intentional use was not clear.* |
| Bite | **1** | Signaller's mouth/teeth close on the recipient’s body - this may be very brief or could be held in place. |
| Bite: threat | **1** | Signaller opens mouth rapidly as if preparing for a bite (often with a movement towards recipient) but moves past or away quickly before making contact. |
| Body cross* | **1** | Signaller is holding arm across their own body (i.e., if the right arm is used it is held across to the left side of the body), either in the air or clutched to the signaller’s chest/front. |
| Bounce | **(1)** | Rhythmic vertical up-down movement (or 'bobbing') of the signaller’s body – in chimpanzees all hands and feet remain on the ground (rarely hands may be free of ground). *There is one clear example* *of a bouncing movement in the mountain gorillas seen performed bipedally before a chest beat (adult female).* |
| Bow | **(1)** | Signaller bends forward from the waist while standing bipedally. Note that head should not make contact with ground, and in typical form body is held straight and only lowered a little (compare: Head stand). *There is one example* *of a bow movement in the mountain gorillas seen performed by an immature individual.* |
| Bump into* | **1** | Signaller approaches (running or slowly walking) recipient and bumps into recipient’s body (full body movement). It is different from “Lean in” as the action is a fast (dynamic) movement and the contact with the recipient very short. |
| Butt | **0** | Signaller's head is briefly and firmly pushed into the body of the recipient (contact is typically short). |
| Chest beat | **1** | Signaller’s hands make (alternating) short audible contact with signaller’s chest (always produced while bipedal, rhythmic, long-distance audible chest beat). |
| Chest beat informal | **1** | Signaller’s hands make (alternating) short audible contact with signaller’s chest (rhythm or beats can be irregular, slower, or less formal etc.). Produced bipedally or sitting, lying etc. *Has to include at least 3 hits, otherwise treated as ‘Hit Self’.* *In Byrne et al. 2017 no distinction made between Chest beat and Chest beat Informal (but see ‘Body drum’).* |
| Circle | **0** | Signaller rotates hands around each other (in gorillas like the start of a chest beat but without any contact with chest). *Circling movements are often observed in the build-up phase of (usually immature) chest beats (so before the actual contact hits, maybe as a sort of ‘finding the rhythm’). “Circle” has not been observed as a separate gesture action in this study. In Byrne et al. (2017) called “Disco arms shake” (includes “Circle hands”)* |
| Clap | **0** | Signaller moves both palms towards each other which are brought together with audible contact (may be repeated). |
| Crouch | **0** | Signaller lowers body by bending knees and/or elbows, while maintaining at least three points of contact with ground. *Not observed in this study but see the gesture action Freeze.* |
| Dangle | **1** | Signaller hangs from one or both hands (or feet) from a branch with at least one limb not in contact with branch, some movement of body as a result of hanging. Includes ‘Dangle with shake’ where the signaller produces an additional shake of arm(s) or leg(s). *Dangle was not coded as often as a gesture action because of difficulties to discern it from play actions. It also only ever was performed by immatures, and almost exclusively to initiate play.* |
| Embrace | **1** | Signaller wraps one or both arms/legs around recipient (not necessarily only affiliative). |
| Finger(s) in mouth | **(1)** | Signaller inserts finger(s) - usually palm down - in the mouth of recipient with contact. Signaller makes movement into recipient's mouth - recipient might open or close mouth a little, but not the same as actively moving towards the other individual's hand to bite it. *There is only one example of this gesture action in the mountain gorillas.* |
| Flap | **0** | Flap movement of extremities, typically with one or both legs. Signaller sits with knees bent and opens/ closes one or both legs to side (can be repeated). |
| Fling | **1** | Rapid movement of hand (from wrist) or arm (from elbow or shoulder; rarely with foot or head) away from the signaller’s body, typically towards recipient. Energy of stroke is focused on way out away from signaller, more relaxed on way back. |
| Freeze* | **(1)** | Signaller is approached by the recipient (e.g., the signaller is positioned in the travel path of the recipient) and instead of e.g., moving away or continuing with their activity the signaller ‘freezes’ into a rather unusual posture (e.g., crouching on the belly exposing the back or lying on the back with limbs stretched to the side exposing the belly). |
| Gaze hold(*) | **-** | Signaller looks towards the recipient's face and 'holds' their gaze (moving their head if needed as the recipient moves theirs), holds for >2 seconds. *Termed ‘Look’ in Byrne et al. (2017). Mountain gorillas employ a gesture action that would fit the label ‘Gaze hold’; however, in mountain gorillas the gaze (which is more an intense starring) is accompanied by a stiff body posture and other behavioural markers. It is a very distinctive behaviour and does not resemble ‘peering’ (or ‘Look’). We therefore classified it under the gesture action ‘Gaze stance’.* *While it is reported, there was not enough example data for ‘Gaze hold’ available from other species for a thorough comparison.* |
| Gaze stance* | **1** | Signaller positions body and head towards the recipient’s face, stares at them and ‘holds’ their gaze. The intensive ‘looking’ lasts at least 2 seconds or longer (unlike e.g., a quick attention checking). The gaze is accompanied by a stiff (motionless) body posture (standing or crouching in front of the recipient), and so is different from the Gaze hold. *See discussion.* |
| Grab | **1** | The hand or foot is firmly closed over part of the recipient’s body or a handful of hair. |
| Grab hold | **1** | Same as "Grab" but hand(s)/foot of signaller stays closed around recipient’s body for >2 seconds. |
| Head avert* | **1** | Signaller’s body is positioned towards the recipient’s body but the head is tilted away from the recipient in an exaggerated almost 90 degree angle. May be interrupted by short monitoring of the recipient’s (re)action (at times this attention checking can resemble the description of a (horizontal) head shake but the movements in the head avert are stiff, whereas the head shake is a relaxed throwing the head in different directions) |
| Head stand | **1** | Signaller bends forward and places head on or very near to ground and pauses, at least briefly, in this position. Contact may be made with recipient’s body. |
| Hit fake* | **1** | The signaller is positioned close to the recipient and both individuals are stationary. The signaller raises the arm/hand and lashes out as if to hit the recipient but does not make contact. The outward and backward movement have the same speed and often the arm/hand is still held up after the first ‘faked’ hit for a while (and the gesture action is then either repeated or the arm slowly put back into a neutral position). If contact is made this gesture action is classified as Hit tap. |
| Hit non-recipient* | **1** | Signaller approaches/positions towards recipient with a young offspring (or a ‘stolen’ infant) clinging to the signaller’s front (belly/chest) and (very) softly hits on the infants back similar to a ‘chest beat’ (so alternating rhythmical hand movements on the clinging infant) while gaze and body posture clearly indicate that the behaviour is directed at the other individual (recipient). Note that very soft (rhythmical) hitting on young infants is also observed in mother-offspring interaction. |
| Hit object/ground | **1** | Signaller makes a short hard contact with the ground/object; energy in movement is focused on the way out, contact can be brief or remain in place for longer. Typically occurs in front of or to the side of the signaller’s body (rather than straight up and down as in Stomp). Produced with a variety of body parts - typically hand, fingers, knuckles, fist, foot. |
| Hit object/ground (soft) | **0** | See “Hit object/ground” but contact is gentle (as in tap for example). *No obvious variation in hit intensity observed in the* *mountain gorillas.* |
| Hit object/ground with object | **1** | An object is brought into short hard contact with another object or the ground. |
| Hit recipient | **1** | See “Hit object/ground”, but signaller makes deliberate contact with the recipient as part of action. |
| Hit recipient (soft) | **1** | See “Hit object/ground (soft)” but contact is deliberately made with the recipient’s body. *There is clear variation in hit intensity for the gesture action ‘Hit recipient’ in the mountain gorillas. This variation was not observed in the two other very common ‘hit’ gesture actions (hit self and hit object ground).* |
| Hit recipient with object | **(1)** | An object is brought into short hard contact with the body of the recipient. *There are two relatively clear observations in this study.* |
| Hit self | **1** | See “Hit object/ground”, but signaller makes deliberate contact with own body as part of action. |
| Hit self (soft) | **0** | See Hit soft object/ground but contact is deliberately made with signaller's body. *No obvious variation in hit intensity observed in the mountain gorillas..* |
| Hit tap* | **1** | The signaller is positioned close to the recipient and both individuals are stationary. The signaller raises the hand, lashes out and makes (very) short contact with the recipient’s body. The outward and backward movement have the same speed and often the arm/hand is still held up after the first ‘tap’ for a while (and the gesture action is then either repeated or the arm slowly put back into a neutral position). In movement form similar to ‘Hit fake’ but there is contact made. |
| Individual move* | **(1)** | Signaller grabs an individual and drags it along the ground as if it was an object. If this was directed at the individual being dragged and not effective in achieving the goal, the action was coded as Pull. However, there were a few occasions where the signaller seemingly just ‘used’ the individual he/she dragged as an object, while directing gaze and other behavioural markers at a different individual. *As this behaviour was only observed a few times and generally difficult to analyse we did not include it in the repertoire.* |
| Jab* | **1** | Signaller makes a short hard contact with the recipient’s body, typically with foot (occasionally with fist), unlike in “Hit recipient” the energy is rapid on both the way to and from the recipient. |
| Jab at* | **(1)** | See Jab - same action but signaller deliberately avoids making contact with the recipient's body. |
| Jump | **1** | While bipedal both feet leave the ground simultaneously, while quadrupedal all four limbs free of ground simultaneously; accompanied by horizontal displacement. |
| Kiss | **1** | Gentle contact with the mouth that doesn't hold the recipient’s body (see "Bite"). Includes the ‘share air’ kiss type where they breath on each other while almost touching. |
| Knock object | **0** | Signaller's knuckles (or rarely heel) brought into short hard audible contact with an object like a tree buttress to make a clear sound. |
| Lay on* | **1** | The signaller lifts their leg to the side (like a dog wanting to urinate) and lays it on the recipient’s body. Depending on the size difference between signaller and recipient this can look quite acrobatic. |
| Leaf clipping | **0** | Signaller tears strips from leaf (or leaves), or across leaves, held with the hand/teeth using the teeth or hands to tear; produces conspicuous sound. |
| Leaf clipping: leaf drop | **0** | Leaf or leaves are plucked off stem with signaller's fingers or mouth and quietly dropped. May be pulled/torn at petiole. Note that there is little tearing action on the leaves as seen in leaf clipping. *Not observed in this study but see ‘Object drop’ gesture action.* |
| Lean in* | **1** | The signaller approaches the recipient and leans into the recipient’s body (usually the signaller’s shoulder or head to the recipient’s chest). It is a contact gesture but if it happens during locomotion, contact may not be stable throughout the duration of the gesture. |
| Locomote bipedal | **1** | Signaller stands bipedally and takes at least one step with each foot. |
| Locomote gallop | **1** | An exaggerated running movement where the limbs movements are typically stiff (straightened) and rhythmic. |
| Locomote recipient* | **1** | Signaller finishes a locomote gesture (bipedal, gallop, stiff walk) by moving over the body of the recipient; includes some contact. |
| Locomote stiff run | **1** | An exaggerated walking movement with stiff limbs and forelimbs held in a straightened position (running) |
| Locomote stiff walk | **1** | An exaggerated walking movement with stiff limbs and forelimbs held in a straightened position (slow locomotion) |
| Lunge | **1** | Signaller’s body is rapidly thrust towards recipient. No contact is made. |
| Object clamp* | **(1)** | Signaller takes an object and carries it by clamping it between body parts. *There is only one example (here the signaller clamped a stick between the head and his chest).* |
| Object drop(*) | **1** | The signaller takes an object (e.g., a stick or moss) and drops it close to (or onto) the recipient. *Potentially the same gesture action as leaf drop in bonobos – not included as new* |
| Object in mouth | **1** | Signaller holds an object (e.g., a small branch) in mouth. Hands should not normally be involved. *In the mountain gorillas this includes attached objects.* |
| Object move | **1** | Object is displaced, contact is maintained through movement. While movement may not be in a single direction it does not include the rapid back and forth of ‘Object move: shake’. Object is free to move and not attached to environment (e.g., a fallen branch). *Note that in rare cases a third-party may be used as the ‘object’ - communication should still be clearly directed to the recipient (so this should not be a triadic interaction).* |
| Object move: fiddle* | **1** | Signaller grabs or touches vegetation in front of the recipient and ‘fiddles’ with it. Object (usually the leaves of a sapling, or the sapling itself) movements look uncoordinated (i.e., going into various directions with no clear pattern) but are calm and slow. Movements usually come out of the hand/wrist or fingers. |
| Object move: shake | **1** | Repeated back and forth movement of an object (typically one still attached in the environment, e.g., sapling). Object movement must be controlled by the signaller’s hand/foot actively shaking the object and not a biproduct of the flexibility of the object. |
| Object on head | **(1)** | Signaller places detached object on their head and leaves it in place. Hand may remain in contact with object for balance. *In mountain gorillas one occasion of this gesture action was observed but the object was attached.* |
| Object stance* | **1** | Signaller grabs a tree trunk or liana usually above shoulder height and leans into it (almost as if stretching). The body is stiff and the posture held in place while there is only little movement of the object that supports it (if any at all, unlike object move). |
| Out | **0** | Typically, the signaller's arm is extended out from the shoulder to the side of the body, elbow and hand are held in line. |
| Over stance | **1** | Signaller pauses while standing with at least one limb that has been moved into position and held over the recipient’s body. In full form signaller’s body forms a bridge over the recipient. |
| Pivot(*) | **1** | Signaller grabs an object (e.g., a small tree trunk or a firm branch) and swings his body in a semicircle around the object’s axis. |
| Place on object/ground* | **1** | Arm is extended in front of the body and hand (or fingers, knuckles) placed on an object or the ground in a location between the signaller and recipient (its usually the ground, but there may be thick vegetation/ or a log in that place). The movement of the arm/hand towards the object/ground is coordinated, the contact may be relatively short or last longer than 2 seconds*. Note that this is not the same gesture action as Touch object, a gesture action that is reported for chimpanzees and bonobos in the context of food sharing (where signallers touch the desired object).* |
| Poke | **0** | Short firm contact made with the signaller’s fingers held straight and ‘pushed’ briefly into the recipient’s body. *Could be confused with ‘Hit tap’* |
| Present | **1** | Body or body part moved to deliberately expose an area to the recipient’s attention (excludes present fingers/hand/toes/foot in a ‘reach’ form). Contact is sometimes made during this gesture. |
| Pull | **1** | Same as “Push” but the force is exerted away from the recipient’s body (not effective in achieving the goal). Usually involves a “Grab” from the hand/foot that is not coded as a separate gesture.  “Pull” vs. “Pull (directed)”: the direction of the movement afterwards is not consistent with the direction of the pull. |
| Push | **1** | Contact with recipient’s body (typically hand or foot) and the force is exerted into the recipient’s body (not effective in achieving the goal). “Push” vs. “Push (directed)”: the direction of the movement afterwards is not consistent with the direction of the push. |
| Raise | **1** | Raise body part (typically hand or arm) in a generally vertical movement, often with a brief pause near the top of the movement. |
| Rake object/ground | **1** | Signaller holds hands with fingers stiff and spread in order to displace objects towards their body, e.g., dry leaves. *In the mountain gorillas performed before hit ground.* |
| Rake self | **0** | Signaller holds hands in distinctive position with fingers stiff and spread and pulls them over their body (e.g., over head, may be repeated; coded as a single gesture unless a clear break/change in movement). Rarely seen - often in association with temper 'tantrums'. |
| Reach | **1** | Body part (typically arm/hand) extended towards the recipient (or in their direction) with no contact. |
| Rocking: bipedal | **0** | Signaller moves body back and forth or side to side while standing bipedally. |
| Rocking: sitting | **0** | Signaller moves body back and forth, or side to side, while sitting (includes single/half 'Rock' movement where the signaller moves back and holds). |
| Roll over | **1** | Signaller rolls or rocks so that their back is on the object/ground exposing their stomach and holds position. |
| Rub | **1** | Signaller pushes/rubs body part up and down against body of recipient (typically with hands or genitals). |
| Shake | **1** | Signaller moves part of their body quickly and repeatedly back and forth (typically hand/arm or head). Movement is loose and typical form has several repetitions. |
| Spin: pirouette | **1** | Signaller stands and turns around their body’s vertical axis while (at times) also displacing along the ground. All spin gesture actions need to include a full 360 degrees of turn (movement is more than would be required for locomotion). |
| Spin: side roulade | **1** | Signaller is lying down and turns around their body’s vertical axis while also displacing along the ground. All spin gesture actions need to include a full 360 degrees of turn (movement is more than would be required for locomotion). |
| Spin: somersault | **(1)** | Signaller’s body is curled into a compact position on the ground and rolled forward or backward so the feet are brought over the head and returned to sitting position. All spin gesture actions need to include a full 360 degrees of turn (movement is more than would be required for locomotion). *Behaviour generally present but spin somersault was only used twice as a potentially communicative signal.* |
| Stance bipedal | **1** | Signaller moves into bipedal stance and holds position (must not be in order to achieve other action, e.g., standing to see something arriving). *Observed over 3 times in this study; however, only few cases and only immatures – unless more data in the future, maybe exclude.* |
| Stiff stance | **1** | Signaller holds stiff quadrupedal body position with stiff limbs and forelimbs (often accompanied by ‘tight lips’ facial expression). |
| Stomp object/ground | **1** | The foot (or sometimes hand) is lifted vertically and brought into short hard contact with the ground (or object). Usually this is the surface that the signaller is standing/sitting on. Typically, with sole of foot/flat or hand/fist. |
| Stomp recipient | **0** | See ‘Stomp object/ground’ but contact is deliberately made on recipient's body. Note that this typically occurs only when standing/sitting on top of someone, otherwise consider if Hit recipient a better fit. *Not observed.* |
| Stroke | **1** | Active gentle movements of the signaller’s palm and/or fingers (rarely other) on the recipient’s body. May include movements in more than one direction. |
| Swing | **1** | Smooth continuous motion of body part (normally arm of leg) back and forth (may be repeated). |
| Throw object | **1** | Object is moved and released so that there is displacement of the object through the air after moment of release. |
| Throw threat | **0** | Object is lifted into position to throw it but is held in that position (typically raised above shoulder) without release. *There is one possible observation of a throw threat in the mountain gorillas, while it was a communicative behaviour, it was unclear whether the initial plan was to throw the object.* |
| Thrust | **0** | Groin is pushed forward towards recipient (may be repeated). |
| Touch | **1** | Light contact (typically of the fingers, knuckles, hand, or foot, rarely other) on the body of the recipient, contact <2 seconds. |
| Touch long | **1** | Same as ‘Touch’ but contact is held for 2 seconds or longer. *Termed ‘Hand(s) On’ in Byrne et al. (2017). In some studies, ‘Hands on’ is only referring to touches on the head (e.g., Genty et al. 2009; Pika et al. 2003; Luef & Liebal 2013)* |
| Touch object | **0** | Same as ‘Touch’ but contact is made with a specific object, usually food (begging/food sharing context). *See comment for ‘Place on object/ground’.* |
| Turn* | **1** | Signaller gazes back at the recipient and then makes an exaggerated half-turn with his/her body and head (one fore- and/or hindlimb may be raised during the turn). Similar to Beckon, however unlike Beckon, the movement is around the signallers own body axis. |
| Water splash | **(1)** | Signaller's limb (typically hand) is moved vigorously through the water so that there is audible displacement of the water. *Bwindi mountain gorilla immatures often play with water/mud, however only observed once as a possible gesture action in a play invitation.* |
| Wave | **0** | Large loose back and forth movements of the signaller's arms while raised above the shoulder. |

**Figure S3. Detection asymptote for mountain gorilla morphs.** When plotting the identified repertoire units (here gesture morphs) against the number of observations (gesture tokens), visual assessment of the asymptote allows us to estimate how likely we have been able to fully describe the morph repertoire (given the number of modifiers and levels included in the morph detection). The morph repertoire appears to be fully catalogued by around n = 1395 gesture tokens.

| 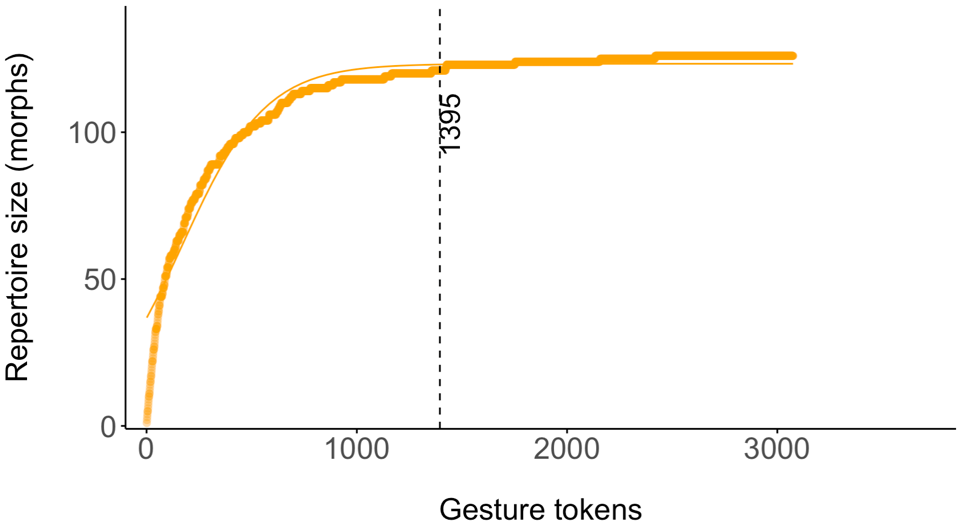 |
| --- |

**Table S12. Specification of n = 126 mountain gorilla morphs.** In the construction of the morphs we applied the latent class analysis (LCA) approach developed by Mielke et al. (2024) and included four modifiers: *Body part*, *Contact recipient*, *Lateral use,* and *Repetition* (see Table S7). The latent class analysis was conducted on 94% of the original dataset (n = 3024 instances; all tokens with unclear modifier levels (n = 97) and gesture actions that had < 10 observations (n = 18 gesture actions; n = 99 tokens) were excluded).

**Table S13. Instance contribution to the repertoire: comparing the most frequent morphs and gesture actions.** The table lists morphs (left side) and gesture actions (right side) by their token contribution (Token contr. (%)). The cumulative contribution (Cumul. contr. (%)) denotes the summed percentages of token contribution of morphs/gesture actions to the data (in order). If contribution was random, each morph would be expected to contribute n = 24.4 tokens to the data, and here each gesture action would be expected to contribute n = 48.8 (n = 3075 gesture tokens in the final morph dataset (post morph detection)). The deviation from the average (Dev. average) indicates how much each morph or gesture action deviated from the respective mean baseline value.

| Morph | n tokens | Token  contr. (%) | Cumul.  contr.  (%) | Dev. average | Gesture action | n tokens | Token  contr. (%) | Cumul.  contr.  (%) | Dev. average |
| --- | --- | --- | --- | --- | --- | --- | --- | --- | --- |
| Chest beat informal.1_1 | 144 | 4.7 | 4.7 | 120 | Push | 251 | 8.2 | 8.2 | 202 |
| Object move.1_3 | 130 | 4.3 | 8.9 | 106 | Grab | 196 | 6.4 | 14.6 | 147 |
| Gaze stance.1_1 | 125 | 4.1 | 13.0 | 101 | Present | 196 | 6.4 | 21.0 | 147 |
| Reach.1_1 | 123 | 4.1 | 17.0 | 99 | Touch | 177 | 5.8 | 26.8 | 128 |
| Stiff stance.1_1 | 115 | 3.8 | 20.7 | 91 | Object move | 153 | 5.0 | 31.8 | 104 |
| Locomote stiff walk.1_1 | 83 | 2.7 | 23.4 | 59 | Chest beat informal | 144 | 4.7 | 36.5 | 95 |
| Touch.1_4 | 81 | 2.7 | 26.0 | 57 | Hit object | 141 | 4.6 | 41.1 | 92 |
| Grab.1_5 | 77 | 2.5 | 28.5 | 53 | Gaze stance | 125 | 4.1 | 45.2 | 76 |
| Grab.2_5 | 72 | 2.4 | 30.8 | 48 | Reach | 123 | 4.0 | 49.2 | 74 |
| Embrace.1_3 | 62 | 2.0 | 32.8 | 38 | Pull | 116 | 3.8 | 53.0 | 67 |
| Present.1_4 | 62 | 2.0 | 34.8 | 38 | Stiff stance | 115 | 3.7 | 56.7 | 66 |
| Pull.1_3 | 56 | 1.8 | 36.6 | 32 | Hit recipient | 110 | 3.6 | 60.3 | 61 |
| Pull.2_3 | 54 | 1.8 | 38.4 | 30 | Locomote stiff walk | 83 | 2.7 | 63.0 | 34 |
| Push.1_8 | 55 | 1.8 | 40.2 | 31 | Embrace | 79 | 2.6 | 65.6 | 30 |
| Hit object.1_4 | 52 | 1.7 | 41.9 | 28 | Grab hold | 73 | 2.4 | 68.0 | 24 |
| Present.2_4 | 52 | 1.7 | 43.6 | 28 | Raise | 60 | 2.0 | 70.0 | 11 |
| Present.4_4 | 52 | 1.7 | 45.3 | 28 | Touch long | 58 | 1.9 | 71.9 | 9 |
| Grab hold.1_2 | 49 | 1.6 | 46.9 | 25 | Bite threat | 46 | 1.5 | 73.4 | -3 |
| Hit recipient.1_6 | 48 | 1.6 | 48.5 | 24 | Hit self | 44 | 1.4 | 74.8 | -5 |
| Raise.1_2 | 50 | 1.6 | 50.1 | 26 | Hit recipient soft | 42 | 1.4 | 76.2 | -7 |
| N = 106 remaining morphs with each < 2% instance contribution to the repertoire | | | | | N = 43 remaining gesture actions with each < 2% instance contribution to the repertoire | | | | |
|  | | | | | | | | | |

The comparison shows that the most commonly used gesture units are of quite different types. For example, the most frequently used gesture action *Push*, appears for the first time as the 14^th^ most frequently used unit in the morph ranking (as it was split into n = 8 separate morphs) and the second most frequently used gesture action *Grab* first occurs at ranks 8 and 9 in the morph units (*Grab* was split into n = 5 separate morphs). *Present* (n = 4 morphs) and *Touch* (n = 4 morphs) also dropped to ranks 11 and 7 respectively, whereas *Object move* (n = 3 morphs) rose from rank 5 to rank 2.

**Figure S4. Relationship between individual repertoire size and gesture tokens in Bwindi mountain gorillas** (n = 27 signallers, n = 2877 gesture tokens; n = 60 gesture actions, n = 2 social units (Bit, Muk)).


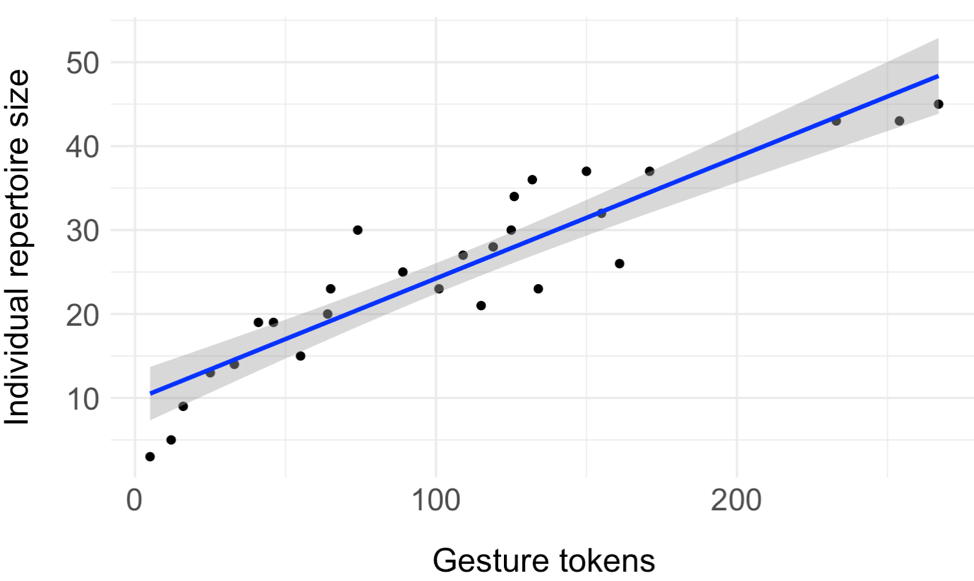


**Table S14. Individual repertoire size and gesturing rate across maturation categories** (n = 27 signallers, n = 2877 gesture tokens; n = 60 gesture actions, n = 2 social units (Bit, Muk)). Mat. Cat-1: *infants* (0-3 years, both sexes); Mat. Cat-2: *juveniles* (> 3 – 6 years, both sexes), Mat. Cat-3: *subadults* (> 6 – 8 years, both sexes) *+ young nulliparous females* (females 8 > 12 years without infants) *+ blackbacks* (males: > 8 – 12 years); Mat. Cat-4: *adults* (males > 12 years; parous females; adult nulliparous females > 12 years).

| **Mat.** | **mean IRS_1** | **mean IRS_3** | **mean IGR_obs** | **mean IGR_vid** | **tokens** | **sgn** |
| --- | --- | --- | --- | --- | --- | --- |
| ***cat-1*** | **13.2** (sd ± 10.2; r = 3-29) | **4.3** (sd ± 3.8; r = 1-9) | **0.36** (sd ± 0.29, r = 0.04-0.88) | **1.6** (sd ± 1.3, r = 0.26-4) | 207 | 6* |
| ***cat-2*** | **32.4** (sd ± 10.0; r = 21-45) | **14.6** (sd ± 7.1; r = 6-22) | **0.85** (sd ± 0.48, r = 0.38-1.35) | **4.5** (sd ± 2.15, r = 0.2-6.8) | 712 | 5 |
| ***cat-3*** | **27.6** (sd ± 10.0; r = 11-43) | **12.7** (sd ± 8.9; r = 4-26) | **0.89** (sd ± 0.46, r = 0.35-1.64) | **8.3** (sd ± 6.82, r = 3.8-23.14^a^) | 692 | 7 |
| ***cat-4*** | **21.1** (sd ± 7.2; r = 5-32) | **9.9** (sd ± 5.4; r = 1-18) | **0.41** (sd ± 0.21, r = 0.05-0.73) | **3.6** (sd ± 1.37, r = 1.03-5.83) | 1266 | 15 |
| Mat. Cat = maturation category; mean IRS_1 = mean individual repertoire size including single gesture action use; mean IRS_3 = mean individual repertoire size with a cut-off at 3 tokens per gesture action and signaller; mean IGR_obs = mean individual gesturing rate (gesture tokens produced by individual per h of focal group observation time); mean IGR_vid = mean individual gesturing rate (gesture tokens produced by individual per h of video footage with individual present); tokens = number of gesture instances; sgn = number of signallers in maturation category; sd = standard deviation, r = range. ^a^ | | | | | | |

**Figure S5. Individual gesturing rates (*IGR_obs*) across sex and maturation classes** (n = 27 signallers, n = 2877 gesture tokens; n = 60 gesture actions, n = 2 social units (Bit, Muk)). Box plots showing individual gesturing rates (n = 33 data points) across four maturation classes: a) irrespective of sex (*cat-1*: infants; *cat-2*: juveniles, *cat-3*: subadults, blackbacks, nulliparous females; *cat-4:* adults) and b) differentiating males and females within each class. Note that 6 out of 27 signallers contributed to more than one maturation class in this comparison. Abbreviations: saf = subadult female; nf = nulliparous female; sam = subadult male; bb = blackback; af = adult female; am = adult male.


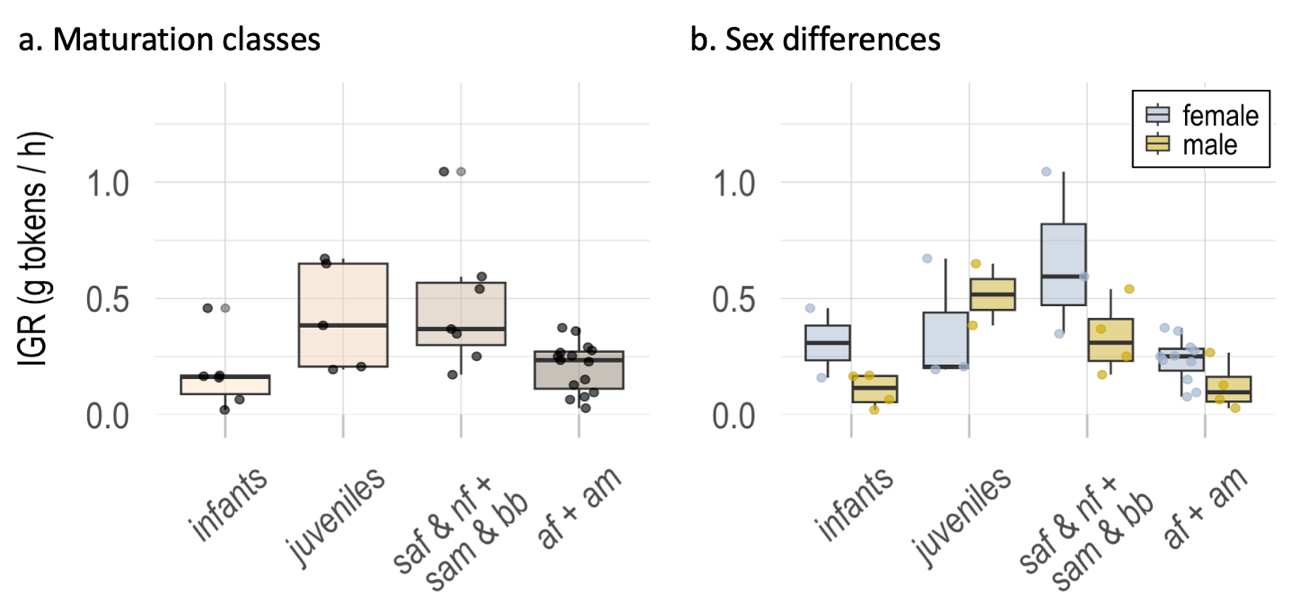


**Table S15. Female and male IRS and IGR across maturation classes** (n = 27 signallers, n = 2877 gesture tokens; n = 60 gesture actions, n = 2 social units (Bit, Muk)). Mat. Cat-1: *infants* (0-3 years, both sexes); Mat. Cat-2: *juveniles* (> 3 – 6 years, both sexes), Mat. Cat-3: *subadults* (> 6 – 8 years, both sexes) *+ young nulliparous females* (females 8 > 12 years without infants) *+ blackbacks* (males: > 8 – 12 years); Mat. Cat-4: *adults* (males > 12 years; parous females; adult nulliparous females > 12 years).

|  | **cat-1** | | **cat-2** | | **cat-3** | | **cat-4** | |
| --- | --- | --- | --- | --- | --- | --- | --- | --- |
|  | ***females***  n = 2 signallers; n = 91 tokens | ***males***  n = 4 signallers; n = 116 tokens | ***females***  n = 3 signallers; n = 397 tokens | ***males***  n = 2 signallers; n = 315 tokens | ***females***  n = 3 signallers; n = 305 tokens | ***males***  n = 4 signallers; n = 387 tokens | ***females***  n = 11 signallers; n = 1059 tokens | ***males***  n = 4 signallers; n = 207 tokens |
| **mean IRS_1** | **16** (sd ± 18.4, r = 3-29) | **11.8** (sd ± 7.4, r = 3-20) | **30.3** (sd ± 12.9, r = 21-45) | **35.5** (sd ± 6.4, r = 31-40) | **27** (sd ± 14, r = 11-37) | **28** (sd ± 11.2, r = 19-43) | **24** (sd ± 5.3, r = 14-32) | **13.5** (sd ± 6.6, r = 5-21) |
| **mean IRS_3** | **5** (sd ± 5.7, r = 1-9 | **4** (sd ± 3.6, r = 1-9 | **13** (sd ± 8.2, r = 6-22 | **17** (sd ± 7.1, r = 12-22 | **14** (sd ± 9.5, r = 4-23 | **11.8** (sd ± 9.7, r = 4-26 | **11.3** (sd ± 4.5, r = 4-18 | **6.3** (sd ± 6.7, r = 1-16 |
| **mean IGR_obs** | **0.66** (sd ± 0.31, r = 0.44-0.88) | **0.21** (sd ± 0.34, r = 0.15-0.33) | **0.71** (sd ± 0.55, r = 0.38-1.35) | **1.05** (sd ± 0.42, r = 0.75-1.35) | **1.19** (sd ± 0.49, r = 0.68-1.64) | **0.67** (sd ± 0.34, r = 0.35-1.11) | **0.47** (sd ± 0.19, r = 0.16-0.73) | **0.24** (sd ± 0.19, r = 0.06-0.52) |
| **mean IGR_vid** | **2.78** ( sd± 1.68, r = 1.59-3.97) | **1.01** ( sd± 0.6, r = 0.26-1.5) | **3.72** ( sd± 2.4, r = 2.19-6.48) | **5.7** ( sd± 1.52, r = 4.62-7.8) | **12.3*** ( sd± 9.6, r = 4.74-23.14) | **5.22** ( sd± 1.63, r = 3.82-7.57) | **3.9** ( sd± 1.0, r = 1.9-5.83) | **2.7** ( sd± 1.93, r = 1.03-5.44) |
| mean IRS_1 = mean individual repertoire size including single gesture action use; mean IRS_3 = mean individual repertoire size with a cut-off at 3 tokens per gesture action and signaller; mean IGR_obs = mean individual gesturing rate (gesture tokens produced by individual per h of focal group observation time); mean IGR_vid = mean individual gesturing rate (gesture tokens produced by individual per h of video footage with individual present); tokens = number of gesture instances; sd = standard deviation, r = range. | | | | | | | | |

**Table S16. N = 15 newly identified, potentially mountain gorilla specific, gesture actions – primary context of use and potential matching descriptions in the gorilla gesture literature.**

| **Newly defined gesture action** | **Definition** | **PBC** | **Potentially matching description in (captive) western lowland gorilla gesture studies (Tanner & Byrne 1999; Pika et al. 2003; Genty et al. 2009)** |
| --- | --- | --- | --- |
| **Body cross** | Signaller is holding arm across their own body (i.e., if the right arm is used it is held across to the left side of the body), either in the air or clutched to the signaller’s chest/front. | Sexual | *no matching description* |
| **Bump into** | Signaller approaches (running or slowly walking) recipient and bumps into recipient’s body (full body movement). It is different from “Lean in” as the action is a fast (dynamic) movement and the contact with the recipient very short. | Miscellaneous | **Genty et al. 2009**: potentially reflected in the *Pounce* gesture type (definition: ‘jumping forward on another individual’) – see also the mountain gorilla gesture action ‘Locomote recipient’ (this study) |
| **Gaze stance** | Signaller positions body and head towards the recipient’s face, stares at them and ‘holds’ their gaze. The intensive ‘looking’ lasts at least 2 seconds or longer (unlike e.g., a quick attention checking). The gaze is accompanied by a stiff (motionless) body posture (standing or crouching in front of the recipient), and so is different from the Gaze hold. | Sexual | **Genty et al. 2009**: potentially a combination of *Look* (definition: ‘staring intensely at another individual’) *+ Stiff stance* (definition: ‘standing rigidly with stiff limbs and forelimbs held tight, facial expression of tight lips usually occurs in sexual context’)  Note: the behaviour depicted in the available example videos did not resemble the mountain gorilla *Gaze stance*, and the *Look* was closer to descriptions of peering (cf. Schuppli et al. 2016)  **Behaviour reported in other studies** *captive western lowland gorillas* (Atsalis et al. 2004; Stoinski et al. 2009), *Virunga mountain gorillas* (Watts 1991; Yamagiwa 1992; Nadler 1989) |
| **Head avert** | Signaller’s body is positioned towards the recipient’s body but the head is tilted away from the recipient in an exaggerated almost 90 degree angle. May be interrupted by short monitoring of the recipient’s (re)action (at times this attention checking can resemble the description of a (horizontal) head shake but the movements in the head avert are stiff, whereas the head shake is a relaxed throwing the head in different directions) | Miscellaneous | **Genty et al. 2009:** a similar behaviour is mentioned in the description of the *Stiff walk* gesture type (definition: ‘walking with rigid forelegs and usually head tilted on the side’)  Note: the head tilt was not coded separately and the equivalent *Locomote stiff* in the mountain gorillas was not frequently accompanied by the gesture action *Head avert* (although *Head avert* was typically accompanied by a stiff body posture). |
| **Hit fake** | The signaller is positioned close to the recipient and both individuals are stationary. The signaller raises the arm/hand and lashes out as if to hit the recipient but does not make contact. The outward and backward movement have the same speed and often the arm/hand is still held up after the first ‘faked’ hit for a while (and the gesture action is then either repeated or the arm slowly put back into a neutral position). If contact is made this gesture action is classified as Hit tap. | Sexual | *no matching description* |
| **Hit non-recipient** | Signaller approaches/positions towards recipient with a young offspring (or a ‘stolen’ infant) clinging to the signaller’s front (belly/chest) and (very) softly hits on the infants back similar to a ‘chest beat’ (so alternating rhythmical hand movements on the clinging infant) while gaze and body posture clearly indicate that the behaviour is directed at the other individual (recipient). Note that very soft (rhythmical) hitting on young infants is also observed in mother-offspring interaction. | Affiliation | *no matching description* |
| **Hit tap** | The signaller is positioned close to the recipient and both individuals are stationary. The signaller raises the hand, lashes out and makes (very) short contact with the recipient’s body. The outward and backward movement have the same speed and often the arm/hand is still held up after the first ‘tap’ for a while (and the gesture action is then either repeated or the arm slowly put back into a neutral position). In movement form similar to ‘Hit fake’ but there is contact made. | Miscellaneous | *no matching description* |
| **Jab** | Signaller makes a short hard contact with the recipient’s body, typically with foot (occasionally with fist), unlike in “Hit recipient” the energy is rapid on both the way to and from the recipient. | Miscellaneous | **Genty et al. 2009**: resembles some (but not all) of the example videos for *Punch* (definition: ‘Hitting another individual forcefully and singly with fist or wrist’) |
| **Lay on** | The signaller lifts their leg to the side (like a dog wanting to urinate) and lays it on the recipient’s body. Depending on the size difference between signaller and recipient this can look quite acrobatic. | Miscellaneous | *no matching description* |
| **Lean in** | The signaller approaches the recipient and leans into the recipient’s body (usually the signaller’s shoulder or head to the recipient’s chest). It is a contact gesture but if it happens during locomotion, contact may not be stable throughout the duration of the gesture. | Affiliation | *no matching description* |
| **Locomote recipient** | Signaller finishes a locomote gesture (bipedal, gallop, stiff walk) by moving over the body of the recipient; includes some contact. | Miscellaneous | **Genty et al. 2009**: potentially reflected in the *Pounce* gesture type (definition: ‘jumping forward on another individual’) – see also the mountain gorilla gesture action ‘Bump into’ (this study) |
| **Object move: fiddle** | Signaller grabs or touches vegetation in front of the recipient and ‘fiddles’ with it. Object (usually the leaves of a sapling, or the sapling itself) movements look uncoordinated (i.e., going into various directions with no clear pattern) but are calm and slow. Movements usually come out of the hand/wrist or fingers. | Sexual | *no matching description* |
| **Object stance** | Signaller grabs a tree trunk or liana usually above shoulder height and leans into it (almost as if stretching). The body is stiff and the posture held in place while there is only little movement of the object that supports it (if any at all, unlike object move). | Sexual | *no matching description* |
| **Place on object/ground** | Arm is extended in front of the body and hand (or fingers, knuckles) placed on an object or the ground in a location between the signaller and recipient (its usually the ground, but there may be thick vegetation/ or a log in that place). The movement of the arm/hand towards the object/ground is coordinated, the contact may be relatively short or last longer than 2 seconds*.* | Sexual | *no matching description* |
| **Turn** | Signaller gazes back at the recipient and then makes an exaggerated half-turn with his/her body and head (one fore- and/or hindlimb may be raised during the turn). Similar to Beckon, however unlike Beckon, the movement is around the signallers own body axis. | Miscellaneous | *no matching description* |

**References**

Atsalis, S., Margulis, S. W., Bellem, A., & Wielebnowski, N. (2004). Sexual behavior and hormonal estrus cycles in captive aged lowland gorillas (*Gorilla gorilla*). *American Journal of Primatology*, *62*(2), 123-132. <https://doi.org/10.1002/ajp.20010>

Byrne, R. W., Cartmill, E., Genty, E., Graham, K. E., Hobaiter, C., & Tanner, J. (2017). Great ape gestures: Intentional communication with a rich set of innate signals. *Animal Cognition*, 20(4), 755–769. <https://doi.org/10.1007/s10071-017-1096-4>

Genty, E., Breuer, T., Hobaiter, C., & Byrne, R. W. (2009). Gestural communication of the gorilla (*Gorilla gorilla*): repertoire, intentionality and possible origins. Animal cognition, 12(3), 527-546. <https://doi.org/10.1007/s10071-009-0213-4>

Grund, C., Badihi, G., Graham, K. E., Safryghin, A., & Hobaiter, C. (2023). GesturalOrigins: A bottom-up framework for establishing systematic gesture data across ape species. *Behavior research methods*, *56*(2), 986-1001. <https://doi.org/10.3758/s13428-023-02082-9>

Grund, C., Robbins, M. M., & Hobaiter, C. (2024). Mountain gorilla gesture action example videos [Data set]. Zenodo. <https://doi.org/10.5281/zenodo.13341597>

Mielke, A., Badihi, G., Graham, K. E., Grund, C., Hashimoto, C., Piel, A. K., ... & Hobaiter, C. (2024). Many morphs: Parsing gesture signals from the noise. *Behavior research methods*, 1-18. <https://doi.org/10.3758/s13428-024-02368-6>

Nadler, R. D. (1989). Sexual initiation in wild mountain gorillas. *International Journal of Primatology*, 10, 81-92. <https://doi.org/10.1007/BF02736246>

Pika, S., Liebal, K., & Tomasello, M. (2003). Gestural communication in young gorillas (*Gorilla gorilla*): gestural repertoire, learning, and use. American Journal of Primatology: Official Journal of the American Society of Primatologists, 60(3), 95-111. <https://doi.org/10.1002/ajp.10097>

Stoinski, T. S., Perdue, B. M., & Legg, A. M. (2009). Sexual behavior in female western lowland gorillas (*Gorilla gorilla gorilla*): evidence for sexual competition. *American Journal of Primatology: Official Journal of the American Society of Primatologists*, *71*(7), 587-593. <https://doi.org/10.1002/ajp.20692>

Tanner, J. E., & Byrne, R. W. (1999). The development of spontaneous gestural communication in a group of zoo-living lowland gorillas. In *The Mentalities of Gorillas and Orangutans: Comparative Perspectives* (pp. 211–239). Cambridge University Press. <https://doi.org/10.1017/CBO9780511542305.012>

Watts, D. P. (1991). Mountain gorilla reproduction and sexual behavior. *American journal of Primatology*, *24*(3‐4), 211-225. <https://doi.org/10.1002/ajp.1350240307>

Yamagiwa, J. (1992). Functional analysis of social staring behavior in an all-male group of mountain gorillas. *Primates*, *33*(4), 523–544. <https://doi.org/10.1007/BF02381153>
